# Type I arginine methyltransferases are intervention points to unveil the oncogenic Epstein-Barr virus to the immune system

**DOI:** 10.1093/nar/gkac915

**Published:** 2022-11-09

**Authors:** Gaelle Angrand, Alicia Quillévéré, Nadège Loaëc, Van-Trang Dinh, Ronan Le Sénéchal, Rahima Chennoufi, Patricia Duchambon, Marc Keruzoré, Rodrigo Prado Martins, Marie-Paule Teulade-Fichou, Robin Fåhraeus, Marc Blondel

**Affiliations:** Institut National de la Santé et de la Recherche Médicale UMR1078; Université de Bretagne Occidentale, Faculté de Médecine et des Sciences de la Santé; Etablissement Français du Sang (EFS) Bretagne; CHRU Brest, Hôpital Morvan, Laboratoire de Génétique Moléculaire, 22 avenue Camille Desmoulins, F-29200 Brest, France; Institut National de la Santé et de la Recherche Médicale UMR1078; Université de Bretagne Occidentale, Faculté de Médecine et des Sciences de la Santé; Etablissement Français du Sang (EFS) Bretagne; CHRU Brest, Hôpital Morvan, Laboratoire de Génétique Moléculaire, 22 avenue Camille Desmoulins, F-29200 Brest, France; Institut National de la Santé et de la Recherche Médicale UMR1078; Université de Bretagne Occidentale, Faculté de Médecine et des Sciences de la Santé; Etablissement Français du Sang (EFS) Bretagne; CHRU Brest, Hôpital Morvan, Laboratoire de Génétique Moléculaire, 22 avenue Camille Desmoulins, F-29200 Brest, France; Institut National de la Santé et de la Recherche Médicale UMR1078; Université de Bretagne Occidentale, Faculté de Médecine et des Sciences de la Santé; Etablissement Français du Sang (EFS) Bretagne; CHRU Brest, Hôpital Morvan, Laboratoire de Génétique Moléculaire, 22 avenue Camille Desmoulins, F-29200 Brest, France; Institut National de la Santé et de la Recherche Médicale UMR1078; Université de Bretagne Occidentale, Faculté de Médecine et des Sciences de la Santé; Etablissement Français du Sang (EFS) Bretagne; CHRU Brest, Hôpital Morvan, Laboratoire de Génétique Moléculaire, 22 avenue Camille Desmoulins, F-29200 Brest, France; Chemistry and Modelling for the Biology of Cancer, CNRS UMR9187 - Inserm U1196, Institut Curie, Université Paris-Saclay, Orsay, Campus universitaire, Bat. 110, F-91405, France; Chemistry and Modelling for the Biology of Cancer, CNRS UMR9187 - Inserm U1196, Institut Curie, Université Paris-Saclay, Orsay, Campus universitaire, Bat. 110, F-91405, France; Institut National de la Santé et de la Recherche Médicale UMR1078; Université de Bretagne Occidentale, Faculté de Médecine et des Sciences de la Santé; Etablissement Français du Sang (EFS) Bretagne; CHRU Brest, Hôpital Morvan, Laboratoire de Génétique Moléculaire, 22 avenue Camille Desmoulins, F-29200 Brest, France; ISP, INRAE, Université de Tours, UMR1282, Tours, Nouzilly, France; Chemistry and Modelling for the Biology of Cancer, CNRS UMR9187 - Inserm U1196, Institut Curie, Université Paris-Saclay, Orsay, Campus universitaire, Bat. 110, F-91405, France; Cibles Thérapeutiques, Institut National de la Santé et de la Recherche Médicale UMR1162, Institut de Génétique Moléculaire, Université Paris 7, Hôpital St. Louis, 27 rue Juliette Dodu, F-75010 Paris, France; RECAMO, Masaryk Memorial Cancer Institute, Zluty kopec 7, 65653 Brno, Czech Republic; Institut National de la Santé et de la Recherche Médicale UMR1078; Université de Bretagne Occidentale, Faculté de Médecine et des Sciences de la Santé; Etablissement Français du Sang (EFS) Bretagne; CHRU Brest, Hôpital Morvan, Laboratoire de Génétique Moléculaire, 22 avenue Camille Desmoulins, F-29200 Brest, France

## Abstract

The oncogenic Epstein-Barr virus (EBV) evades the immune system but has an Achilles heel: its genome maintenance protein EBNA1. Indeed, EBNA1 is essential for viral genome maintenance but is also highly antigenic. Hence, EBV seemingly evolved a system in which the glycine–alanine repeat (GAr) of EBNA1 limits the translation of its own mRNA to the minimal level to ensure its essential function, thereby, at the same time, minimizing immune recognition. Therefore, defining intervention points at which to interfere with GAr-based inhibition of translation is an important step to trigger an immune response against EBV-carrying cancers. The host protein nucleolin (NCL) plays a critical role in this process via a direct interaction with G-quadruplexes (G4) formed in the GAr-encoding sequence of the viral EBNA1 mRNA. Here we show that the C-terminal arginine–glycine-rich (RGG) motif of NCL is crucial for its role in GAr-based inhibition of translation by mediating interaction of NCL with G4 of EBNA1 mRNA. We also show that this interaction depends on the type I arginine methyltransferase family, notably PRMT1 and PRMT3: drugs or small interfering RNA that target these enzymes prevent efficient binding of NCL on G4 of EBNA1 mRNA and relieve GAr-based inhibition of translation and of antigen presentation. Hence, this work defines type I arginine methyltransferases as therapeutic targets to interfere with EBNA1 and EBV immune evasion.

## INTRODUCTION

The Epstein-Barr virus (EBV) is the first oncogenic virus described in human (1–3). EBV is linked to at least 1% of cancers worldwide (4), which include Burkitt's and Hodgkin's lymphomas, nasopharyngeal carcinoma, 10% of gastric cancers and potentially gliomas (5–7). Like the other gamma herpesviruses, EBV is a latent virus that evades the host immune system. However, EBV presents an Achilles heel: its virally encoded genome maintenance protein EBNA1 (Epstein-Barr nuclear antigen 1), which is both essential for the virus (for the maintenance of its genome) and highly antigenic. In addition, infected individuals may contain T cells raised against EBNA1 (8–10). Hence, EBV evolved a mechanism by which EBNA1 self-inhibits the translation of its own mRNA, thereby minimizing the production of EBNA1-derived antigenic peptides (11,12). Although not fully elucidated, this mechanism critically involves the Gly–Ala repeat (GAr) of EBNA1 which is encoded by a guanine-repeat-containing mRNA sequence that was shown to be able to form clusters of up to thirteen G-quadruplexes (G4) that have been involved in GAr-based inhibition of translation (13). G4 are non-canonical secondary structures that may assemble in guanine-rich DNA or RNA. G4 are formed by the stacking of at least two G-quartets which consist of a planar arrangement of four guanines connected by Hoogsteen hydrogen bonds and stabilized by a central cation, most often K^+^. G4 structures within G-rich DNA or RNA have been implicated in gene regulation where they can affect transcription, splicing or translation (14–19). Recently, the host cell nucleolin protein (NCL in human, Nsr1 in the budding yeast *Saccharomyces cerevisiae*) has been shown to directly mediate EBV immune evasion through binding to G4 of the GAr-encoding sequence of EBNA1 mRNA (20,21). Indeed, the binding of NCL to G4 of EBNA1 mRNA is required for GAr-based inhibition of both translation and antigen presentation. Importantly, this protein–RNA-G4 interaction is a relevant intervention point to interfere with EBNA1 immune evasion, as several G4 ligands, which include the benchmark compound PhenDC3 as well as the new derivatives PyDH2 and PhenDH2, are able to interfere with both this interaction and, consequently, with GAr-based limitation of translation and antigen presentation (21,22). Hence G4 that form in the GAr-encoding sequence of EBNA1 mRNA constitute a recognition platform for the binding of the host cell NCL, a mechanism which is critically involved in EBNA1 immune evasion. However, several important questions remain such as about the precise role of NCL in GAr-based translation inhibition, or the identification of the domain of NCL that is involved in binding of G4 of EBNA1 mRNA.

**Figure 1.**
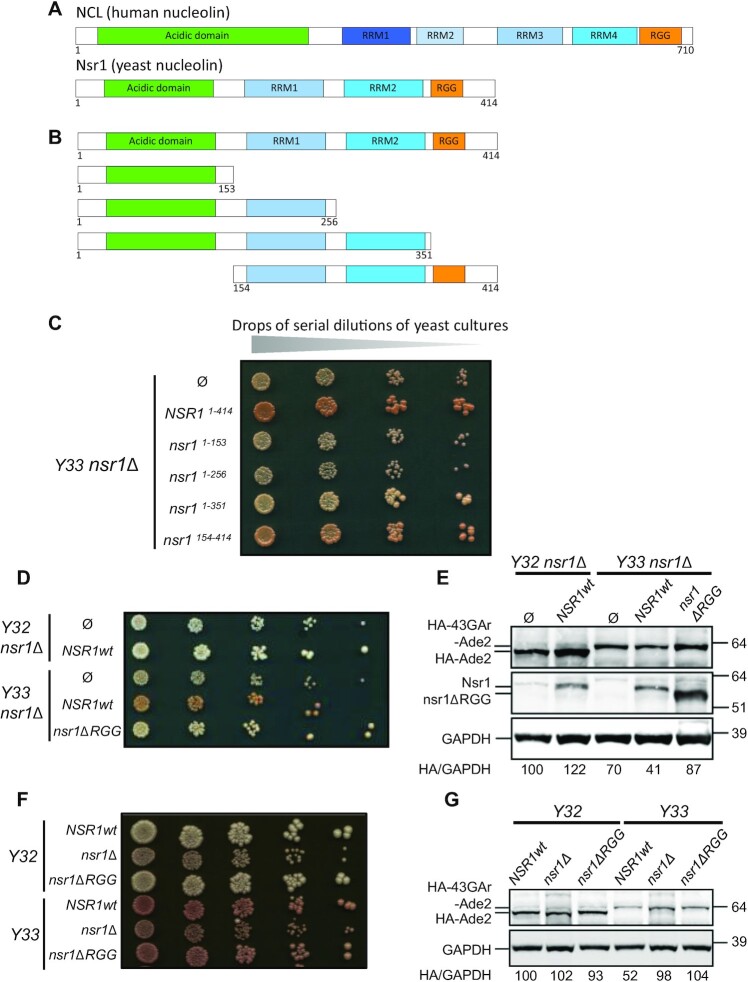
The C-terminal arginine–glycine rich (RGG) motif of yeast nucleolin Nsr1 is necessary for its role in GAr-based inhibition of translation. (**A**) Schematic representation of the various domains of the human (NCL) and yeast (Nsr1) nucleolin. Note that the two proteins share the same organization, in particular central RRMs (four for NCL and only two for Nsr1 which are probably orthologues of RRM3 and 4, hence the colour code) and the C-terminal RGG motif. Both RRM and RGG are known as RNA-binding motifs. (**B**) Schematic representation of the various constructions used for the domain analysis of the yeast nucleolin Nsr1. (**C**) Analysis of the ability of these various constructs to complement the role of Nsr1 in GAr-based inhibition of translation in a *Y33 nsr1Δ* strain, as compared with the wild-type Nsr1. Serial dilutions of the various transformed strains were spotted onto agar-based solid medium. The colour of yeast colonies is used as the readout. Note that all the constructs that lack the C-terminus that contains the RGG motif are unable to complement the deletion of the *NSR1* gene, as indicated by the white colour of the yeast colonies. (**D**) Ability of a form of yeast nucleolin deleted of its C-terminal RGG motif (nsr1ΔRGG) to complement the role of Nsr1 in GAr-based inhibition of translation in a *Y33 nsr1Δ* strain as compared with the wild-type Nsr1. Briefly, a yeast strain that expresses HA-43GAr-Ade2 or, as a control, HA-Ade2 as sole source of Ade2, and deleted for the *NSR1* gene was transformed with either an empty vector (negative control), or a plasmid allowing expression of full-length Nsr1wt (positive control), or of nsr1ΔRGG, as indicated. Serial dilutions of the various transformed strains were spotted onto agar-based solid medium and the colour of the resulting transformed strains was assessed. A full level of expression of HA-Ade2 leads to the formation of white colonies, whereas the reduced level of HA-43GAr-Ade2 due to the GAr-based inhibition of translation leads to pink colonies. (**E**) Western blot analysis of the level of Ade2 or HA-43GAr-Ade2 in the same strains. HA-Ade2/GAPDH or HA-43-GAr-Ade2/GAPDH ratios are indicated below the gels. (**F**) *Y33 nsr1ΔRGG*, a yeast strain in which only the sequence encoding the RGG motif was deleted from the endogenous chromosomal *NSR1* gene, displays the same pink phenotype as *Y33 nsr1Δ* regarding GAr-based inhibition of translation but grows like a *Y33 NSR1wt* strain, indicating that nsr1ΔRGG is functionally expressed. (**G**) Western blot analysis of the level of Ade2 or HA-43GAr-Ade2 in the same strains. HA-Ade2/GAPDH or HA-43-GAr-Ade2/GAPDH ratios are indicated below the gels.

NCL is a multifunctional nucleolar DNA/RNA-binding protein widely conserved among eukaryotes, whose major role is in RNA metabolism, in particular rRNA maturation (23–25). NCL also plays additional roles in chromatin remodelling, cell cycle control, transcription and apoptosis. NCL is one of the first G4-interacting proteins identified (26). One of its best documented mechanisms of action on G4 is its role as a repressor of transcription through stabilization of DNA-G4 in the c-Myc promoter (27). Later, NCL was shown to recognize DNA-G4 within the long terminal repeat (LTR) promoter of human immunodeficiency virus (HIV), thereby silencing the provirus transcription (28). More recently, in addition to its role in the immune evasion of EBV through binding to G4 of EBNA1 mRNA (21), NCL has been involved in suppressing hepatitis C virus (HCV) replication through binding to the viral core RNA-G4 structure (29). NCL is ubiquitous and is therefore likely to interact at many G4 loci of DNA or RNA. The structural determinants of G4–NCL interactions are not precisely known but, recently, a strong preference of NCL for binding G4-DNA harbouring a long central loop has been reported by several groups (30–32). NCL is composed of three main domains: (i) the N-terminal domain that contains four acidic stretches; (ii) the central region that contains four tandem RNA recognition motifs (RRM1–RRM4); and (iii) the arginine/glycine/glycine (RGG) motif at the C-terminus. Importantly, most nucleolin homologues known to date share the same organization, with some variations in the number of RRMs; for example, the yeast nucleolin Nsr1 contains only two RRMs but possesses the acidic N-terminal domain as well as the C-terminal RGG motif (Figure 1A). RGG motifs represent low complexity sequences which can lead to disordered regions (33) and have been involved in interaction with nucleic acids or various proteins (34).

Methyl groups can be attached to the nitrogen atoms of arginine within polypeptides, a process termed arginine methylation. This post-translational modification is conducted by protein arginine methyltransferases (PRMTs) (35). PRMTs catalyse the transfer of a methyl group from *S*-adenosylmethionine (SAM) to the guanidino nitrogen atoms of arginine, resulting in the formation of methylarginine and *S*-adenosylhomocysteine. Arginine can be methylated in three different ways on its guanidino group: it can be monomethylated, symmetrically dimethylated or asymmetrically dimethylated, each of which has potentially different functional consequences (36). There are nine PRMTs in human that are divided into three groups: type I, type II and type III (37). PRMT7 is the single type III PRMT and only catalyses the formation of monomethylarginine (MMA), whereas both type I and type II PRMTs catalyse the formation of MMA first, and then, from this intermediate, of dimethylarginine (DMA). Starting from MMA, type I PRMTs (PRMT1, PRMT2, PRMT3, PRMT4, PRMT6 and PRMT8) catalyse the formation of asymmetric dimethylarginine (aDMA), whereas type II PRMTs (PRMT5 and PRMT9) catalyse the formation of symmetric dimethylarginine (sDMA). Arginine methylation occurs on a variety of protein sequence motifs, but the RGG motifs are the most commonly reported (38,39) and, in budding yeast, it is estimated that the majority of arginine methylation occurs in RGG motifs (40). This is in line with the fact that RGG is the canonical motif substrate established for Hmt1p, a type I PRMT and the main budding yeast PRMT, which is responsible for 66% of arginine monomethylation and 89% of asymmetric dimethylation of intracellular proteins in *S. cerevisiae* (41).

Here, we have first determined that the C-terminal RGG motif of nucleolin is essential for both its ability to bind G4 of EBNA1 mRNA and its role in GAr-based inhibition of translation. This is true for both Nsr1, the yeast nucleolin, and for NCL, the human nucleolin. We also show that the ability of nucleolin to interact with G4 of EBNA1 mRNA, as well as the GAr-based limitation of both translation and antigen presentation, depend on type I PRMTs, in particular on PRMT1 and PRMT3.

Hence, the RGG motif of the host protein NCL is critically involved in the binding of G4 of the viral EBNA1 mRNA, and this interaction depends on type I arginine methyltransferases that thus represent relevant therapeutic targets to interfere with EBNA1 and EBV immune evasion.

## MATERIALS AND METHODS

### Yeast strains, genetic manipulation and culture media

All the strains used in this study are derived from the W303a *WT* K699 strain (42): *MAT*a, *leu2-3*, 112 *trp1-1*, *can 1-100*, *ura3-*1, *ade2-1*, *his3-11, 15*.

***Y32**: MAT a, leu2-3, 113 trp1-1, can 1-100, ura3-1, ade2-1:: his5 ^S.pombe^, his3-11,15, met15::HA-ade2*

***Y33**: MAT a, leu2-3, 113 trp1-1, can 1-100, ura3-1, ade2-1:: his5 ^S.pombe^, his3-11,15, met15::HA-43GAr -ade2*

***Y32 nsr1Δ**: MAT a, leu2-3, 113 trp1-1, can 1-100, ura3-1, ade2-1:: his5 ^S.pombe^, his3-11,15, met15::HA-ade2, nsr1::KANMX6*

***Y33 nsr1Δ**: MAT a, leu2-3, 113 trp1-1, can 1-100, ura3-1, ade2-1:: his5 ^S.pombe^, his3-11,15, met15::HA-43GAr-ade2, nsr1::KANMX6*

***Y32 nsr1ΔRGG**: MAT a, leu2-3, 113 trp1-1, can 1-100, ura3-1, ade2-1:: his5 ^S.pombe^, his3-11,15, met15::HA-ade2, nsr1ΔRGG::KANMX6*

***Y33 nsr1ΔRGG**: MAT a, leu2-3, 113 trp1-1, can 1-100, ura3-1, ade2-1:: his5 ^S.pombe^, his3-11,15, met15::HA-43GAr-ade2, nsr1ΔRGG::KANMX6*

***Y32 hmt1Δ**: MAT a, leu2-3, 113 trp1-1, can 1-100, ura3-1, ade2-1:: his5 ^S.pombe^, his3-11,15, met15::HA-ade2, hmt1::KANMX6*

***Y33 hmt1Δ**: MAT a, leu2-3, 113 trp1-1, can 1-100, ura3-1, ade2-1:: his5 ^S.pombe^, his3-11,15, met15::HA-43GAr-ade2, hmt1::KANMX6*

Yeasts were growth on the following media: YPD (10 g/l yeast extract, 20 g/l peptone, 20 g/l d-glucose) and DO-TRP (6.7 g/l yeast extract without amino acids, 0.74 g of CSM-TRP, 20 g/l d-glucose). For solid media, agar was added at a final concentration of 20 g/l.

### Plasmid construction

All the plasmids were generated using standard procedures. The T4 DNA ligase was obtained from Promega and the restriction enzymes were purchased from New England Biolabs. Plasmids maintenance and amplification were carried out in a chemically competent *Escherichia coli* strain. The plasmids pRS414 containing NSR1 and parts of the coding sequence were constructed as follows: the DNA fragments were amplified by the forward primers 5′-CGCGGATCCATGGCTAAGACTACTAAAGTAAAAGGTAAC-3′ or 5′-GCGCGCGGATCCATGTCTTCCAACAAGAAGCAAAAA-3′, and the reverse primers 5′-CCGCTCGAGCGGTTAATCAAATGTTTTCTTTGAACCAG-3′, 5′-CGGCCGCTCGAGTTACTCTTCGTCTTCTTCTTCTTC-3′, 5′-CGGCCGCTCGAGTTACTTGGCACGATCGTTGTTAC-3′ or 5′-CGGCCGCTCGAGTTAACCATCGTTGTTTGGTCTTGG-3′. The corresponding polymerase chain reaction (PCR) fragments were cloned into BamHI and XhoI cloning sites of the pRS414-ADH vector.

All generated constructions were verified by PCR amplification on clones, restriction enzyme digestion and sequencing.

### Yeast protein extraction

A 4 ml aliquot of exponentially growing yeast of 0.7–0.8 OD_600 nm_ was harvested, washed in 1× TE, pelleted and then suspended into 300 μl of lysis buffer [25 mM Tris–HCl pH 6.8, 10% glycerol, 5% β-mercaptoethanol, 5% sodium dodecylsulphate (SDS), 8 M urea, 0.02% bromophenol blue)

### Cell culture and transfection

H1299 cells are derived from metastatic lymph node from lung carcinoma. Mutu-1 cells are derived from an EBV-positive Burkitt lymphoma. Raji cells are from a type III latency EBV-infected Burkitt's lymphoma. B3Z T cells are from a hybridoma expressing a T-cell receptor (TCR) that specifically recognizes ovalbumin (OVA; 257–264: SIINFEKL) in the context of H-2Kb. H1299, Mutu-1 and Raji cells were cultured in RPM1-1640 supplemented with 10% foetal bovine serum and 2 mM l-glutamine. B3Z cells were cultured in the same medium supplemented with 50 μM β-mercaptoethanol. Transfections were performed using GeneJuice^®^ transfection reagent (Merck), or by electroporation using the Amaxa Cell Line Nucleofector^®^ kit V (Lonza) for Raji and Mutu-1 cells, in both cases according to the manufacturer's protocol.

### Small interfering RNA (siRNA)-mediated knockdown

A total of 50 000 cells per well were seeded in 6-well plates and transfected the following day with 0.75 μg of EBNA1 or EBNA1ΔGAr expression vectors using Genejuice according to the manufacturer’s protocol (Merck). Transfected cells were treated with the indicated concentration of control siRNA or FlexiTube GeneSolution for PRMT 1, 3 and 5 (Qiagen). siRNAs were implemented according to the manufacturer’s protocol using HiPerFect transfection reagent (Qiagen). Cells were collected for western blot analysis 48 h after expression vector transfection.

### Protein extraction from mammalian cells

Whole cells of 75–90% confluence in 6-well plates were harvested, washed in 1× phosphate-buffered saline (PBS) and suspended in lysis buffer [20 mM HEPES pH 7.5, 50 mM β-glycerolphosphate, 1 mM EDTA pH 8, 0.5 mM Na_3_VO_4_, 100 mM KCl, 10% glycerol, 1% Triton, anti-protesase cocktail (Roche 11697498001)]. These cell suspensiosn were mechanically lysed before centrifugation at 16 000 *g* for 20 min at 4°C, and the protein concentration was determined using the Bradford assay.

### Western blotting

Equal protein quantities and volumes of all samples were loaded and run on Bolt or NuPAGE (polyacrylamide gel electrophoresis) 10% Bis-Tris Protein gels (Invitrogen) then transferred onto a 0.45 μM nitrocellulose membrane (GE Healthcare). Membranes were blocked in 1× PBS, 0.1% Igepal and 3% bovine serum albumin (BSA), and incubated with the indicated primary antibodies: mouse anti-Actin (Abcam ab3280, 1/5000), mouse anti-glyceraldehyde phoshate dehydrogenase (GAPDH; Abcam ab125247, 1/5000), mouse anti-EBNA1 (Cytobarr OTX1-EBNA1, 1/2000), rat anti-haemagglutinin (HA; Roche 11867423001, 1/2000), rabbit anti-NCL (Abcam ab70493, 1/5000), mouse anti-NSR1 (Abcam ab4642, 1/5000), rabbit anti-PRMT1 (Merck 07-404, 1/2000), rabbit anti-PRMT5 (Merck 07-405, 1/2000) and rabbit anti-PRMT3 (Abcam ab191562, 1/10000). The membranes were then washed with fresh 1× PBS, 0.1% Igepal and incubated with the indicated secondary antibodies, conjugated to horseradish peroxidase: rabbit anti-mouse (Dako P0161, 1/3000), swine anti-rabbit (Dako P0217, 1/2000) and goat anti-rat (Millipore AP136P, 1/3000). The membranes were washed again and analysed by enhanced chemiluminescence in buffer (Tris-base pH 8.5, 12.5 nM coumaric acid, 2.25 nM luminol and 0.15% H_2_O_2_) using a Vilber-Loumart Photodocumentation Chemistart 5000 imager. All the experiments were repeated at least three times. Relative proteins levels for each sample were normaliazed to GAPDH or Actin protein levels as indicated, using Image J software.

### T-cell proliferation assay

A total of 50 000 H1299 cells were seeded in 6-well plates and co-transfected the following day with 0.5 μg of Kb expression vector and 0.25 μg of 235GAr-OVA, OVA or control plasmids using Genejuice according to the manufacturer’s protocol (Merck). Forty-eight hours later, the transfected cells were seeded in 24-well plates at a density of 1 × 10^5^ in the presence of 1 × 10^5^ cells of the B3Z T hybridoma in 2 ml final volume. After 48 h, the supernatant was withdrawn for an enzyme-linked immunosorbent assay (ELISA) to evaluate the interleukin-2 (IL2) production in order to estimate T-cell activation and therefore antigen presentation. IL2 levels were measured using the IL2 ELISA MAX™ Standard kit (Biolegend, USA) according to the manufacturer's instructions.

### RNA pulldown

#### Yeast protein extracts

A 50 ml aliquot of 0.8–1.0 OD_600 nm_ exponentially growing cells was collected and cell pellets were suspended in 2 ml of lysis buffer [25 mM Tris–HCl, pH 7,4, 100 mM NaCl, 100 mM EDTA 0.1% Triton, antiprotease cocktail (Roche, 11697498001)]. After addition of 450–600 μm glass beads (Sigma-Aldrich, G8772), cells were lysed by six cycles of vortexing for 30 s followed by 30 s ice-cooling, shaken for 2 min at 25 Hz in a mixer mill (Retsch MM400) and centrifuged for 3 min at 800 *g* at 4°C. Supernatants were recovered and protein concentrations were determined using the Bradford assay.

#### Mammalian cell protein extracts

Cells were collected 48 h post-treatment and suspended in 200 μl of lysis buffer [20 mM HEPES, pH 7,5, 50 mM β-glycerophosphate, 1 mM EDTA, pH 8, 0.5 mM Na_3_VO_4_, 100 mM KCl, 10% glycerol, 1% Triton, antiprotease cocktail (Roche, 11697498001)]. Cells were lysed as mentioned above. Supernatants were recovered and protein concentrations were measured using the Bradford assay.

#### RNA pulldown experiments

Yeast extracts or mammalian cell extracts were used for pulldown assays with the following G4-forming oligonucleotides: GQ 5′-GGGGCAGGAGCAGGAGGA-3′-Biotin-TEG, GM 5′-GAGGCAGUAGCAGUAGAA-3′-Biotin-TEG and ARPC2 5′-AGCCGGGGGCUGGGCGGGGACCGGGCUUGU-3′Biotin-TEG. G4 were formed by heating the RNA oligonucleotides at 95°C during 5 min then cooling them down to 4°C at a rate of 2°C/min in the presence of 100 mM KCl to favour G4 formation. To avoid unspecific binding, high-affinity streptavidin–Sepharose beads (GE Healthcare, 28985799) were incubated in 1 ml of blocking buffer [10 mM Tris–HCl, pH 7.5, 100 mM KCl, 0.1 mM EDTA, 1 mM dithiothreitol (DTT), 0.01% Triton, 0.1% BSA, 0.02% *S. cerevisiae* tRNAs (Sigma-Aldrich, 10109495001)] for 1 h at 4°C on a rotating wheel. A 10 μg aliquot of each folded biotinylated RNA oligonucleotide was incubated with 50 μl of solution containing the streptavidin–Sepharose beads for 90 min at 4°C on a rotating wheel. Then 500 μg of cell extracts (mammalian or yeast) or 200 ng of recombinant NCL were treated with 200 U/ml of RNase inhibitor (NEB, M0307S) for 90 min at 4°C on a rotating wheel. These extracts or recombinant NCL were incubated with the RNA oligonucleotides bound to the streptavidin beads for 90 min at room temperature. Beads were then washed five times with lysis buffer and lysis buffer with increasing KCl concentrations (200–800 mM). Proteins still bound to beads after the washes were eluted using 2× loading buffer (2× Laemmli buffer with 5% β-mercaptoethanol) and analysed by western blotting against NCL or Nsr1.

### Proximity ligation assay (PLA)

Cells were fixed in 1× PBS, 4% paraformaldehyde for 20 min and permeabilized for 10 min with 0.4% Triton X-100, 0.05% CHAPS. The EBNA1–digoxigenin probe mRNA (5′-CTTTCCAAACCACCCTCCTTTTTTGCGCCTGCCTCCATCAAAAA-3′) at 50 ng/well was denaturated for 5 min at 80°C. The probe hybridization reaction was carried out in 40 μl of hybridization buffer (10% formamide, 2× SCC, 0.2 mg/ml *E. coli* tRNA, 0.2 mg/ml salmon sperm DNA and 2 mg/ml BSA). The fixed cells were washed and blocked into the blocking solution (1× PBS, 3% BSA, 0.1% saponin) before incubation with the primary antibodies (anti-digoxigenin, Sigma 1/200 and anti-NCL, Abcam 1/1000). The PLA reaction was performed under the manufacturer’s protocol using the Duolink PLA *in situ* kit, PLA probe anti-rabbit plus, PLA probe anti-mouse Minus and the *in situ* detection reagent FarRed, all from Sigma. The results were analysed using a Zeiss Axio Imager M2. All the PLA experiments were performed at least three times independently, and the following controls probes were implemented: without mRNA probe or without primary antibodies.

### RNA extraction and quantitative reverse transcription–PCR (RT–qPCR)

Total H1299 or Mutu-1 cellular RNA was extracted using NucleoSpin^®^ RNA Plus (Macherey Nagel). cDNA synthesis was carried out using 1 μg of RNA and M-MuLV Reverse Transcriptase (NEB) together with random primer. cDNA samples were analysed by qPCR using Master Mix PCR Power SYBR™ Green (Applied Biosystems™). The relative abundance of target mRNA was normalized using GAPDH as an endogenous control. Quantification of gene expression was determined using the 2^−ΔΔCT^ method. The primers used for PCR were GAPDH-forward, 5′-GAGTCAACGGATTTGGTCGT-3′; GAPDH-reverse, 5′-CACAAGCTTCCCGTTCTCAG-3′; EBNA1-forward, 5′-GGCAGTGGACCTCAAAGAAGAG-3′; and EBNA1-reverse, 5′- CAATGCAACTTGGACGTTTTTG-3′. All the experiments were performed in duplicate and repeated twice.

### MTT assay

About 15 000 Mutu-1 cells were plated at 0.1 ml per well in 96-well, flat-bottom plates and exposed to the indicated compounds at the indicated concentrations or dimethylsulphoxide (DMSO; vehicle). After 48 h, 10 μl of MTT solution [5 mg/ml in PBS (pH 7.4), CT01-5, Merck Millipore] was added to each well and cells were incubated for 4 h. A mixture of isopropanol/0.1 N HCl/10% Triton X-100 (0.1 ml) was added to each well to dissolve the formazan crystals, and the absorbance was then measured at 540 nm.

### Immunofluorescence

H1299 cells were plated on 13 mm diameter coverslips in 24-well plates and were transfected with HA-NCL-wt, HA-NCL-R10A or HA-NCL-R10F plasmids. At 24 h post-transfection, cells were fixed with 4% paraformaldehyde in PBS for 20 min and permeabilized with 0.4% PBS, Triton X-100, 0.05% CHAPS for 10 min at room temperature. After incubation in blocking buffer (1× PBS, 3% BSA, 0.1% saponin) for 30 min at room temperature, samples were incubated with a mouse polyclonal anti-HA antibody (a kind gift from Borek Vojtesek, Masaryk Memorial Cancer Institute, Brno, Czech Republic) at 1/1000 for 2 h at room temperature followed by incubation with a 1/500 dilution of the goat anti-mouse immunoglobulin G (IgG) secondary antibody conjugated to Alexa Fluor^®^ 594 (Invitrogen). Both antibodies were diluted in blocking buffer. 4′,6-Diaminidino-2-phenylindole (DAPI) was used for nuclear counterstaining and the images were taken using a Zeiss Axio Imager M2. The experiments were repeated twice.

### NCL methylation

NCL recombinant protein was prepared and purified as previously described (32). Methylation of purified NCL recombinant protein was performed in a buffer containing 50 mM Tris pH 7.5, 1 mM EDTA, 200 mM NaCl. NCL (5 μM) was incubated with 50 nM SAM (Sigma-Aldrich, Saint-Quentin-Fallavier) and 240 μg/ml of recombinant human PRMT1 protein (ab89007, Abcam) at 30°C for 3 h. The conditions of the methylation reaction i.e. time, buffer and stoichiometry of the partners (SAM:NCL:PRMT = 10:1:1), were previously determined through preliminary qualitative experiments using [^3^H]SAM followed by nitrocellulose membrane filtration (Bio-dot). Methylated NCL was purified by using a HiLoad Superdex^®^ column and stored in 50 mM Tris pH 8, 200 mM NaCl buffer at −80°C. Mass spectrometry analysis was performed to identify the number and sites of methylation.

### Electrophoretic mobility shift assay

The annealed EBNA1 and EBNA1-2rep were prepared by mixing 5′-radiolabelled [γ-^32^P]oligonucleotide with non-radiolabelled oligonucleotide (100 nM) in K-100 buffer [10 mM LiCaco2, 100 mM KCl (pH 7.2)], then heating at 95°C for 5 min followed by slow cooling. For protein or peptide binding, the annealed RNAs (final concentration of 20 nM) were incubated with proteins or peptides in the desired concentration range (0–1 μM for proteins and 0–56 nM for peptides) in 1× binding buffer [10 mM Tris–HCl (pH 7.5), 1 mM EDTA, 100 mM KCl, 0,1 mM DTT, 5% glycerol and 0.01 mg/mL BSA] at room temperature for 1 h. The samples were loaded on a 10% or 15% polyacrylamide native gel (37.5:1 acrylamide:bis ratio) for recombinant proteins or peptides, respectively. After electrophoresis, the gels were exposed in a phosphoimager cassette and scanned on a Typhoon Trio Variable mode imager. The quantitative gel was fitted in GraphPad Prism7 with the Hill slope. Representation of means and standard deviation (SD) values are from three independent experiments.

### Sequences of peptides and oligonucleotides for EMSAs

RGG, H-EGGFGGRGGGRGGFGGRGGGRGGRGGFGGRGRGGFGGRGGFRGGRGGGGD-OH

RGG-met; H-EGGFGG(ADMA)GGG(ADMA)GGFGG(ADMA)GGG(ADMA)GG(ADMA)GGFGGADMA)G(ADMA)GGFGG(ADMA)GGFADMA)GG(ADMA)GGGGD-OH (where ADMA is asymmetrically dimethylated arginine); EBNA1, GGG-GCA-GGA-GCA-GGA-GGA; EBNA1-2repeat, GGG-GCA-GGA-GCA-GGA-GGA-GGG-GCA-GGA-GCA-GGA-GGA

Oligonucleotides were purchased from Eurogentec (HPLC purification). Peptides were synthesized by Pepscan (HPLC purification).

### Statistics analyses

Data were analysed by analysis of variance (ANOVA) in conjunction with Tukey's test using GraphPad Prism 5 Software. Data shown are the mean ± SD. *P <0.05; ***P <0.0001; ns, not significant.

## RESULTS

### The C-terminal RGG motif of yeast nucleolin Nsr1 is necessary for its role in GAr-based inhibition of translation and for the ability of yeast and human nucleolin to bind G4 of EBNA1 mRNA

As stated above, Nsr1, the budding yeast (*S. cerevisiae*) nucleolin, shares the same domain organization as NCL, the human nucleolin, apart from the fact that Nsr1 contains only two central RRMs, instead of four in NCL, which probably correspond to RRM3 and 4 of NCL (Figure 1A). In addition, GAr-based inhibition of translation is fully operational in budding yeast (43) where it also involves nucleolin (20). This explains the relevance of the yeast-based genetic screen that led to the identification of nucleolin as the first host cell factor critically involved in GAr-based inhibition of translation (21). This study also shows that human NCL can complement the effect of the loss of yeast Nsr1 on GAr-based inhibition of translation in yeast. Taken together, these results indicate that yeast is a relevant system to model NCL- and GAr-based inhibition of translation. Importantly, and in contrast to NCL in human cells, Nsr1 is not an essential gene, as yeast *nsr1Δ* strains, although growing quite poorly, are viable (21,44). For all these reasons, we decided to use yeast *nsr1Δ* strains to determine the involvement of the various domains of nucleolin in GAr-based inhibition of translation, as this cellular context allows clear-cut situations where the only source of nucleolin is either full-length Nsr1, or parts of it, that were expressed back in yeast. We also made use of the yeast model for GAr-based inhibition of translation which we recently created (43). This model is based on a fusion between a GAr domain of 43 amino acids (43GAr) and the yeast Ade2 reporter protein. Because GAr is able to self-inhibit the translation of its own mRNA in yeast, this leads to a reduction in Ade2 level (43). This can easily be monitored as yeast which express Ade2 at a functional level form white colonies, whereas yeast that do not express Ade2 readily form red colonies, and any intermediate level of Ade2 leads to the formation of pink colonies whose intensity of coloration is inversely proportional to the level of Ade2 expressed. Hence, a yeast strain expressing the *HA-43GAr-ADE2* construct (*Y33* strain) forms pink colonies and exhibits an intermediate level of HA-43GAr-Ade2 protein, whereas a control strain expressing *HA-ADE2* from the same promoter (*Y32* strain) forms white colonies that contain a higher level of HA-Ade2 protein (43). Importantly, the deletion of the *NSR1* gene in the *Y33* strain (hence termed *Y33 nsr1Δ*) led to the formation of white colonies that express a high level of HA-43GAr-Ade2 as, in yeast too, the GAr-based inhibition of translation depends on nucleolin. In contrast, the deletion of the *NSR1* gene had no effect on HA-Ade2 expression in Y32 strains (hence termed *Y32 nsr1Δ*), demonstrating that the effect of nucleolin on protein expression is GAr dependent (21). Starting from the *Y33 nsr1Δ*, we first determined the effect of re-expressing, from a low copy number centromeric plasmid, full-length Nsr1, or various parts of it (Figure 1B), on the GAr-based inhibition of translation, using as readouts the colour of the various yeast strains and the level of expression of 43GAr-Ade2 or Ade2. As shown in Figure 1C and D, as expected, reintroduction of full-length Nsr1 (1–414) led to pink colonies that express a reduced level of HA-43GAr-Ade2, similar to the original *Y33* strain. The same pink colour was obtained when expressing a form of Nsr1 deleted of its N-terminal acidic domain (154–414), indicating that this domain is not required for the involvement of Nsr1 in GAr-based inhibition of translation (Figure 1C). In contrast, the *Y33 nsr1Δ* strain expressing various forms of Nsr1 that all lack the C-terminal end containing the RGG motif [1–351, that lacks only the C-terminal RGG motif (hence termed nsr1ΔRGG in the following); 1–256, that lacks both RRM2 and RGG; and 1–153, that lacks RRM1, RRM2 and RGG] remains white, suggesting that the inhibitory effect of GAr on protein expression was not restored (Figure 1C). This was checked for the *Y32 nsr1Δ* and *Y33 nsr1Δ* expressing nsr1ΔRGG (Figure 1D, E), indicating that the RGG motif is necessary for the crucial role of Nsr1 in GAr-based inhibition of translation. To confirm this result, we deleted only the RGG motif-encoding sequence from the endogenous chromosomal copy of the *NSR1* gene in the *Y33* strain (to yield *Y33 nsr1ΔRGG*), and in the *Y32* strain (to yield *Y32 nsr1ΔRGG*) as a control. As shown in Figure 1F and G, the *Y33 nsr1ΔRGG* strain displays the same phenotype (white colonies expressing a higher level of 43GAr-Ade2) than the *Y33 nsr1Δ* strain regarding GAr-based inhibition of translation. Of note, the *Y33 nsr1ΔRGG* strain grows like the *Y33 NSR1wt* strain, whereas the *Y33 nsr1Δ* strain exhibits a significant growth defect as reported by us and others (21,44). This shows that the RGG motif is necessary for the role of Nsr1 in GAr-based inhibition of translation, but not for its other functions required for an optimal yeast growth, in good agreement with recently published results (45). In contrast, the results shown in Figure 1C suggest that the two RRMs (RRM1 and 2) are necessary for optimal yeast growth of the *Y33 nsr1Δ* strain as only the forms of Nsr1 that lack RRM1 or both RRM1 and 2, in addition to the C-terminal RGG motif, display the same small colony phenotype as the control strain transformed with an empty vector. Importantly, this result also indicates that the defect in GAr-based inhibition of translation of the yeast strains deleted for the *NSR1* gene cannot be attributed to their slow growth phenotype. It also shows that the role of Nsr1 in GAr-based inhibition of translation can be genetically separated from its central role in cell fitness.

**Figure 2.**
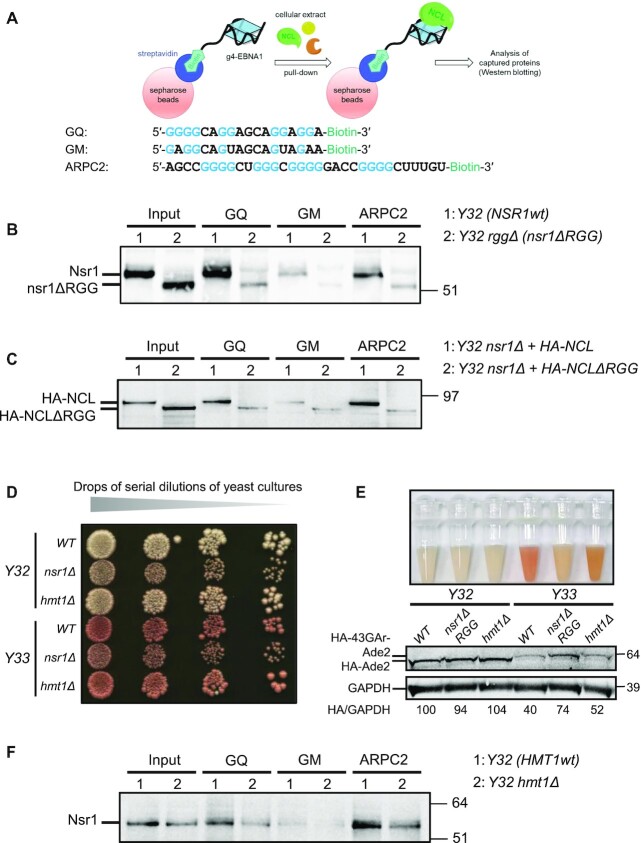
The C-terminal RGG motif of yeast nucleolin Nsr1 is necessary for its binding to G4 of EBNA1 mRNA which also depends on the main yeast type I arginine methyltransferases Hmt1. (**A**) Scheme depicting the principle of the RNA pulldown experiment. The sequence of the various RNA oligonucleotides used is shown and the guanines (G) implicated in the formation G4 are highlighted in blue. GQ: RNA oligonucleotide containing the prototypal repeated sequence which forms the most probable G4 in GAr-encoding sequence of EBNA1 mRNA. GM (negative control): mutated version of GQ in which four guanines were replaced by adenine or uridine to prevent formation of G4. ARPC2 (positive control): RNA oligonucleotide containing the sequence of *ARPC2* mRNA that forms a G4 known to bind NCL (46). (**B**) RNA pulldown of extracts from yeast cells expressing Nsr1wt [1: *Y32* (*NSR1wt*)] or nsr1ΔRGG [2: *Y32 rggΔ* (*nsr1ΔRGG*)] as the sole source of nucleolin. The protein still bound after an 800 mM KCl wash were eluted and analysed by SDS–PAGE and western blot. Like the human nucleolin NCL, the yeast nucleolin Nsr1 binds to G4 of *EBNA1* and *ARPC2* mRNA, and this binding is G4 specific as only residual binding was found on GM beads. In contrast, only residual binding is observed for nsr1ΔRGG. The blot represents *n* ≥3. (**C**) The same experiment as in (B), except that RNA pulldowns were performed using extracts from *nsr1Δ* yeast cells expressing human HA-tagged wild-type nucleolin, HA-NCL (1: *Y32 nsr1Δ* + *HA-NCL*) or a version deleted of its C-terminal RGG motif, NCLΔRGG (2: *Y32 nsr1Δ* + *HA-NCLΔRGG*) as the sole source of nucleolin. In contrast to NCL which binds to G4 of both EBNA1 and ARPC2 in a G4-dependent manner, only residual binding is observed for NCLΔRGG. The blot represents *n* ≥3. (**D**) A similar experiment to that shown in Figure 1C was performed and indicates that the ability of GAr to limit protein expression is lower in an *hmt1Δ* yeast strain (Y33 *hmt1Δ*) as compared with the *HMT1wt* Y33 strain. (**E**) The same yeast strain as in (D) was grown in synthetic liquid medium up to stationary phase and photographed. In addition, western blot analysis of the level of Ade2 or HA-43GAr-Ade2 was performed. HA-Ade2/GAPDH or HA-43-GAr-Ade2/GAPDH ratios are indicated below the gels. The blot represents *n* ≥3. This experiment confirms that the ability of GAr to limit protein expression is less in an *hmt1Δ* yeast strain (Y33 *hmt1Δ*) as compared with the *HMT1wtY33* strain. (**F**) The same RNA pulldown experiment as in (B) and (C) was performed except that the following yeast extracts were used: 1: *Y32* (*HMT1wt*) or 2: *Y32 hmt1Δ*. The binding of the yeast nucleolin Nsr1 on G4 of EBNA1 mRNA is significantly decreased when the *HMT1* gene is absent. The deletion of the *HMT1* gene also affects the binding of Nsr1 on ARPC2 mRNA G4 (positive control).

Because the C-terminal RGG motif of Nsr1 is required for its role in GAr-based inhibition of translation, and as we previously showed that the direct interaction between human nucleolin NCL and G4 of EBNA1 mRNA is required for the GAr-based limitation of both translation and antigen presentation (21), we then compared the ability of the Nsr1 and nsr1Δrgg proteins to interact with G4 of EBNA1 mRNA. For this purpose, we performed RNA pulldown assays as previously described (21,46). Briefly, this assay is based on the use of an RNA oligonucleotide containing a sequence that can form G4, conjugated to biotin, which can therefore be precipitated by magnetic Sepharose beads conjugated to streptavidin (Figure 2A). In this way, proteins which present affinity for the selected RNA-G4 can be precipitated and detected by western blotting. We first determined if yeast nucleolin Nsr1 does bind to G4 of EBNA1 mRNA, as has been shown previously for human nucleolin NCL (21), and if this binding depends on the presence of its C-terminal RGG motif. For this purpose, lysate from *Y32* or *Y32 rggΔ* cells was applied to the following matrices: streptavidin-coupled beads either together with GQ (containing the most probable G4 of the GAr-encoding sequence of EBNA1 mRNA), GM (negative control: same sequence except that four guanines critical for G4 formation were replaced by adenine or uracil) or ARPC2 (positive control containing a G4 present in *ARPC2* mRNA and that has been shown to bind NCL) RNA oligonucleotides (Figure 2A). As shown in Figure 2B, an efficient binding of Nsr1 to the prototypical G4 found in EBNA1 mRNA was observed, which is fully in line with the already described binding of human nucleolin NCL on the same matrix (21). As for NCL, this binding mostly depends on G4 since only a residual binding was observed on GM control that cannot form G4, whereas a significant binding was also observed on ARPC2 matrix (positive control that forms RNA-G4 that binds NCL). Importantly, only a residual binding on GQ, comparable with the binding of Nsr1 on GM, was observed for nsr1Δrgg, whereas Nsr1 and nsr1Δrgg (which were expressed from the *NSR1* endogenous promoter in their normal chromosomal context) were both expressed at a similar level (input, two first lanes). The same result was observed with ARPC2 RNA oligonucleotide, confirming that the binding of yeast nucleolin Nsr1 on RNA-G4 depends on its C-terminal RGG motif.

We then performed the same experiments with human nucleolin NCL. For this purpose, we expressed NCL, or NCLΔRGG, from the constitutive *ADH* promoter in the *Y32 nsr1Δ* strain. We chose to express NCL, or NCLΔRGG, in an *nsr1Δ* yeast strain to ensure that the sole source of nucleolin is the one we expressed. Indeed, in human cells in which NCL is an essential gene whose level of expression cannot be significantly modified, dealing with a mixture of NCL and NCLΔRGG would be inevitable, thereby complicating the analysis. Hence, here again, we made use of yeast genetics and of the fact that nucleolin is not essential in yeast. The *ADH* promoter was chosen because it leads to expression of human nucleolin NCL which is comparable with that of yeast nucleolin Nsr1. As shown in Figure 2C, we obtained the same result with NCL and NCLΔRGG as when using yeast nucleolin Nsr1 and nsr1Δrgg: NCL bound efficiently to the GQ matrix whereas only a residual binding was observed for NCLΔRGG, although it was repeatedly more expressed than NCL in yeast (input, first two lanes). The same result was observed with ARPC2 RNA oligonucleotide, confirming that the binding of human nucleolin NCL on RNA-G4, in particular on G4 of EBNA1 mRNA, also depends on its C-terminal RGG motif.

Hence, we concluded that the binding of both yeast and human nucleolin (Nsr1 and NCL, respectively) on the G4 of the GAr-encoding sequence of EBNA1 mRNA requires their C-terminal RGG motif. The residual binding observed for wild-type Nsr1 and NCL on the GM matrix, or for NCLΔRGG and nsr1Δrgg on the GQ or ARPC2 matrix, is probably due to the RRM domains which possess an intrinsic RNA binding affinity, whereas the RGG motif seems to be crucial for the specific affinity of nucleolin for RNA-G4.

### The main yeast type I arginine methyltransferase Hmt1p is necessary for efficient GAr-based inhibition of translation and binding of nucleolin to G4 of EBNA1 mRNA

In several instances, methylation of RGG motifs has been reported to interfere, positively or negatively, with their ability to interact with other proteins as well as with nucleic acids (34). RGG is the canonical substrate motif established for Hmt1p, a type I PRMT which is the main yeast PRMT and responsible for 66% of arginine monomethylation and 89% of asymmetric dimethylation of intracellular proteins in this model eukaryote. Since it is estimated that, in budding yeast, the majority of arginine methylation occurs on RGG motifs, we tested the impact of inactivating the *HMT1* gene on the GAr-based inhibition of translation and on the ability of Nsr1 to interact with G4 of EBNA1 mRNA. For this, we deleted the *HMT1* gene in both *Y32* and *Y33* strains to yield *Y32 hmt1Δ* and *Y33 hmt1Δ* strains, respectively. We then observed the impact of *HMT1* gene deletion on GAr-based inhibition of protein expression using the same assay as in Figure 1C, D and F. As shown in Figure 2D and E, the *Y33 hmt1Δ* strain presents a whiter phenotype than the control *Y33* strain, a phenotype that is comparable, although less pronounced, with that of the *Y33 nsr1Δ* strain, suggesting that the ability of 43GAr to inhibit expression of 43GAr-Ade2 is partially compromised in the *Y33 hmt1Δ* strain. As we observed no difference in colour in the control strains *Y32* and *Y32 hmt1Δ*, we concluded that the partial loss of GAr-based inhibition of protein expression observed in the *Y33 hmt1Δ* strain, as compared with the *Y33* strain, is GAr dependent. In addition, using western blot analysis, we determined the level of Ade2 and 43GAr-Ade2 proteins in *hmt1Δ* and *nsr1ΔRGG* strains, as compared with wild-type controls in both Y32 and Y33 backgrounds (Figure 2E). We found that 43GAr-Ade2 was slightly more expressed in *Y33 hmt1Δ* as compared with *Y33 HMT1wt*, in good agreement with the slightly whiter phenotype observed on plates (Figure 2D) and in liquid culture (Figure 2E, upper panel). This suggests that modulation of the methylation of the RGG motif of Nsr1 may interfere with its ability to interact with G4 of the GAr-encoding sequence of *EBNA1* mRNA. To test this hypothesis, we performed the same RNA pulldown experiment as described in Figure 2B, except that we compared the ability of Nsr1 extracted from *Y32* or *Y32 hmt1Δ* strains to interact with G4 of EBNA1 mRNA, the G4 of *ARPC2* mRNA (positive control) or the GM RNA oligonucleotide (negative control). As shown in Figure 2F, the ability of Nsr1 to interact with both GQ and ARPC2 RNA oligonucleotides is significantly decreased when Nsr1 is extracted from the *Y32 hmt1Δ* strain as compared with the *Y32* strain. Of note, this decrease is not as strong as that observed in Figure 2B, when comparing the binding of Nsr1 with that of nsr1Δrgg. This is in line with the partial loss of GAr-based inhibition of protein expression observed in the *Y33 hmt1Δ* strain and with the fact that Hmt1 is not the sole PRMT in yeast, which implies that the effect on RGG methylation might only be partial in *hmt1Δ* strains.

We concluded that the requirement of the RGG motif of Nsr1 for the role of this protein in GAr-based inhibition of translation and for its ability to interact with G4 of EBNA1 mRNA in yeast both depend on an optimal type I protein arginine methyltransferase (PRMT) activity.

### Inhibition of human type I protein arginine methyltransferases impacts GAr-based inhibition of protein expression

**Figure 3.**
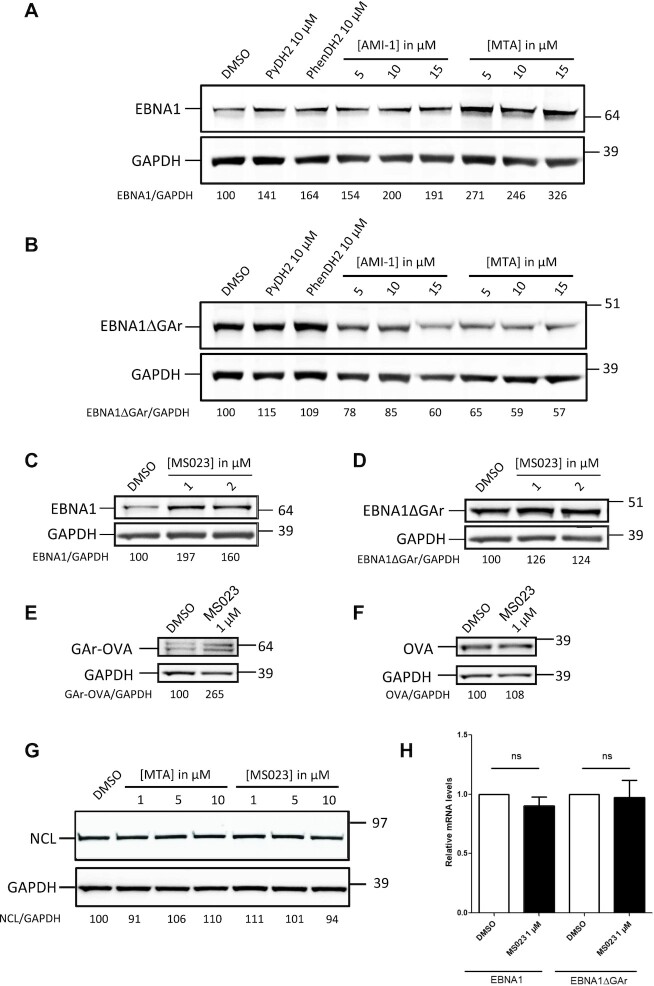
Inhibition of human type I arginine methyltransferases using specific inhibitors impacts GAr-based inhibition translation and has no effect on NCL level. SDS–PAGE and western blot analysis of the level of EBNA1 or EBNA1ΔGAr in response to treatments with various inhibitors of arginine methyltransferases. H1299 cells were transfected with EBNA1- (**A** and **C**) or EBNA1ΔGAr-expressing vectors (**B** and **D**) and treated, or not, with the various drugs, as indicated. GAPDH was used as a loading control. EBNA1/GAPDH or EBNA1ΔGAr/GAPDH ratios are indicated below the gels. The G4 ligands PyDH2 and PhenDH2 were used as positive controls in (A) and (B). SDS–PAGE and western blot analysis of the level of GAr-OVA or OVA in response to treatments with MS023. H1299 cells were transfected with GAr-OVA- (**E**) or OVA-expressing vectors (**F**) and treated, or not, with MS023, as indicated. GAPDH was used as a loading control. GAr-OVA/GAPDH or OVA/GAPDH ratios are indicated below the gels. The NCL steady-state level was determined in H1299 cells treated with various concentrations of MTA or MS023, as indicated (**G**). None of the drugs impacts NCL level. (**H**) Levels of EBNA1 or EBNA1ΔGAr as determined using RT–qPCR.

We next assessed if inhibition of human PRMTs, in particular type I PRMTs (since Hmt1p is a type I PRMT), also affects GAr-based limitation of translation. For this purpose, we first determined the effect of drug- or siRNA-based inhibition of PRMTs, in particular type I PRMTs, on GAr-based limitation of protein expression. Hence, we tested the effect of 7,7′-carbonylbis(azanediyl)bis(4-hydroxynaphthalene-2-sulphonic acid) (AMI-1) or 5′-deoxy-5′(methylthio)adenosine (MTA) molecules on the level of EBNA1 or EBNA1ΔGAr in H1299 cells. AMI-1 was the first identified pharmacological compound targeting endogenous methyltransferases and has been isolated on the basis of its ability to inhibit both yeast and human type I protein arginine methyltransferases Hmt1p and PRMT1 (47). It is generally considered that AMI-1 selectively inhibits type I PRMTs (PRMT1, 3, 4 and 6), but it has been suggested that it could also inhibit PRMT5, the main type II PRMT (48). In contrast, MTA is a natural metabolite that is considered to be a general inhibitor of the three types of PRMTs (49), in particular of PRMT5 (50,51). As shown in Figure 3, treatment of H1299 cells transfected with vectors allowing expression of EBNA1 (Figure 3A), or EBNA1ΔGAr (Figure 3B), by AMI-1 or MTA, led to a GAr-dependent increase in EBNA1 expression which was even more pronounced than that induced by PhenDH2 or PyDH2, two G4 ligands which have been optimized for binding to G4 of EBNA1 mRNA as well as for their ability to outcompete NCL for binding on these G4 (22). These results confirmed those obtained in yeast and indicate that type I PRMTs are important for GAr-based inhibition of protein expression.

To further test this hypothesis, we next examined the effect of MS023, a recently isolated PRMT inhibitor that has been shown to specifically inhibit type I PRMTs (52). MS023 gave similar results to AMI-1 (Figure 3C), whereas it had no noticeable effect on EBNA1ΔGAr (Figure 3D). We have also tested the effect of MS023 on GAr-OVA or OVA proteins as it has been shown that GAr is able to limit expression of OVA. As shown in Figure 3E and F, MS023 strongly increased the expression of GAr-OVA whereas it had no noticeable effect on OVA. As a control, we determined the effect of MTA and MS023 on NCL expression, as a decrease in NCL level upon treatment with these PRMTs inhibitors might readily explain their effect on EBNA1 expression. Importantly, neither MTA nor MS023 had an effect on the NCL protein level (Figure 3G). Finally, we also determined that MS023 had no effect on EBNA1 and EBNA1ΔGAr mRNA levels (Figure 3H; Supplementary Figure S1). Altogether, these experiments confirm that inhibition of type I PRMTs specifically interferes with GAr-based inhibition of protein expression.

**Figure 4.**
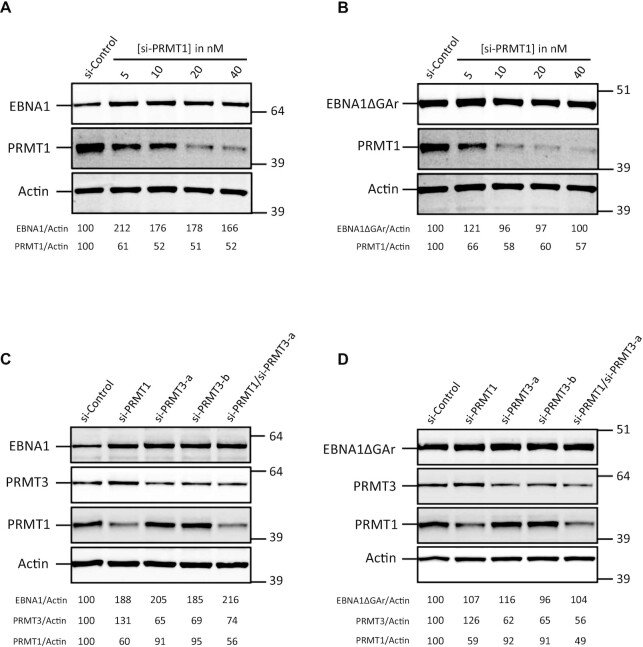
Inhibition of human type I arginine methyltransferases using siRNA impacts GAr-based inhibition of protein expression. SDS–PAGE and western blot analysis of the level of EBNA1 or EBNA1ΔGAr in response to knockdown of various PRMTs. H1299 cells were transfected with EBNA1- (**A** and **C**) or EBNA1ΔGAr-expressing vectors (**B** and **D**) and with control siRNA or siRNA targeting various PRMTs, as indicated. Actin was used as a loading control. EBNA1/Actin or EBNA1ΔGAr/Actin ratios are indicated below the gels. Blots represent *n* ≥3.

We then tested the effect of down-regulating various PRMTs using siRNA. We first determined the effect of down-regulating, independently or together, PRMT1 and PRMT3, the two main type I PRMTs. As shown in Figure 4A and C, down-regulation of PRMT1 alone, or of PRMT3 alone, led to a significant and comparable increase in EBNA1 expression, whereas down-regulation of both PRMT1 and 3 led to a slightly stronger effect. All these effects were GAr specific since no significant effect was observed for EBNA1ΔGAr (Figure 4B, D). We also tested the effect of down-regulating PRMT5, the main type II PRMT, and found that it had no significant effect on EBNA1 expression (not shown). In line with this, we found no additive effect when down-regulating PRMT5 together with PRMT1.

Taken together, all these results confirm that inhibition of type I PRMTs (by drugs or siRNA) interferes with GAr-based inhibition of translation.

### Inhibition of human type I protein arginine methyltransferases impacts the ability of NCL to interact with G4 of EBNA1 mRNA

**Figure 5.**
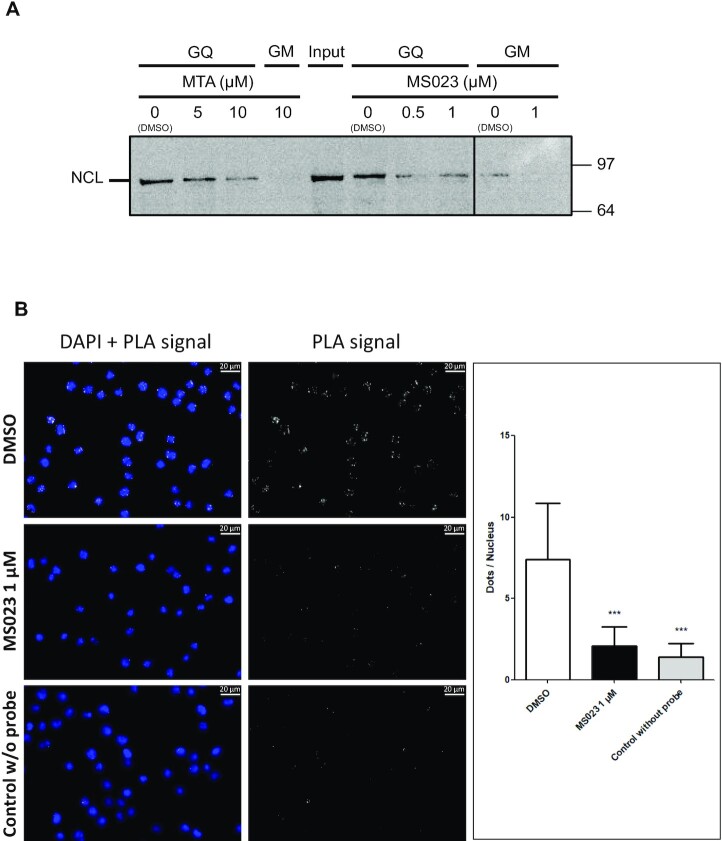
Inhibition of human type I arginine methyltransferases impacts the ability of NCL to interact with G4 of EBNA1 mRNA. (**A**) The same RNA pulldown experiments as in Figure 2 were performed except that extracts from human H1299 cells treated, or not (DMSO control vehicle), with various concentrations of the two PRMT inhibitors MTA or MS023 as indicated were used. Either MTA or MS023 interferes with binding of NCL to G4 of EBNA1 mRNA. (**B**) Adaptation of the PLA to monitor protein–RNA interaction performed in Mutu-1 cells natively expressing EBNA1. Left and middle panels: microscopy images of cells treated with DMSO (compound vehicle, control) or MS023 (1 μM) or negative control cells without probe, as indicated. Nuclei were revealed by DAPI staining and appear in blue; white dots (PLA signals) indicate interaction between NCL and G4 of EBNA1 mRNA. Right panel: number of nuclear PLA signals (dots) per cell in Mutu-1 cells treated with DMSO (control) or with MS023 (1 μM) or in cells of the negative control (without probe). Data from two biological replicates, 200 cells per sample were analysed by ANOVA in conjunction with Tukey's test using GraphPad Prism 5 for Windows (GraphPad Software) (***P <0.0001).

As inhibition of type I PRMTs impacts the GAr-based inhibition of protein expression in both yeast and human cells, we next determined, using RNA pulldown experiments and proximity ligation assay (PLA), if inhibition of PRMTs also interferes with the binding of human nucleolin NCL on G4 of EBNA1 mRNA to confirm the results obtained for yeast nucleolin Nsr1 in Figure 2F. We first performed the same pulldown experiments as in Figure 2 except that, instead of yeast cell lysates, we used cell lysates from human H1299 cells treated with MTA or MS023, or with vehicle (DMSO) as a negative control. As shown in Figure 5A, the treatment with MTA or MS023 decreases in a dose-dependent manner the binding of NCL to G4 of EBNA1 mRNA, whereas it had no significant effect on the steady-state level of NCL (Figure 3G). This result indicates that type I PRMT activity is required for efficient binding of NCL on G4 of EBNA1 mRNA. Next, we wanted to verify if the inhibition of PRMTs affects the NCL–GAr G4 interaction *in cellulo*. Hence, we performed a PLA adapted to protein–RNA interaction (53) to assess if MS023 (Figure 5B) interferes with the binding of endogenous NCL on endogenous EBNA1 mRNA in EBV-infected Mutu-1 cells. As previously reported (21,53), we observed PLA dots mostly in the nucleus, or close to it, in Mutu-1 cells treated with vehicle (DMSO), confirming that the NCL–EBNA1 mRNA interaction essentially takes place in the nucleus or in the cytoplasm in the vicinity of the nucleus. In contrast, treatment with 1 μM MS023 led to a significant decrease in the number of PLA dots per cell, reaching a value comparable with that obtained for the negative control without probe (Figure 5B). Similar results were obtained with treatment with 5 μM MTA (Supplementary Figure S2).

Taken together, these results indicate that type I PRMTs are required, both in yeast and in human cells, for the interaction between nucleolin and G4 of EBNA1 mRNA. As this interaction is crucial for GAr-based inhibition of translation, this readily explains why inactivating type I PRMTs interferes with GAr-based inhibition of translation.

### Changing arginines of the RGG motif of both Nsr1 and NCL to alanines suppresses GAr-based inhibition of protein expression and interaction of NCL with G4 of EBNA1 mRNA, whereas replacement by the bulky hydrophobic phenylalanines mimicking methylated arginines maintains both these phenomenon

**Figure 6.**
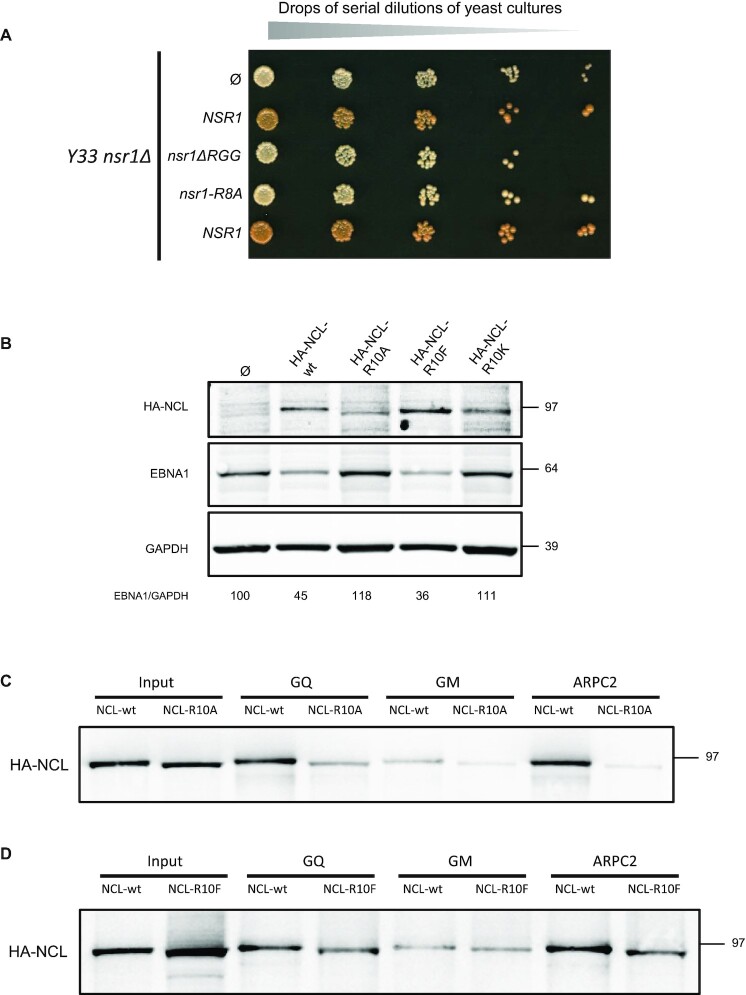
Changing arginines of the RGG motif of both Nsr1 and NCL to alanines suppresses GAr-based inhibition of protein expression and interaction of NCL with G4 of EBNA1 mRNA, whereas changing arginines of NCL to the bulky hydrophobic phenylalanine mimicking methylated arginine maintains both of these phenomena. (**A**) The same experiments as that shown in Figures 1C and 2D were performed and indicate that the eight arginines of the RGG motif of yeast nucleolin Nsr1 are necessary for its function in GAr-based inhibition of protein expression. (**B**) SDS–PAGE and western blot analysis of the level of endogenous EBNA1 in the EBV-infected Raji cell line overexpressing wild-type or mutated versions of HA-tagged nucleolin (HA-NCL-wt, HA-NCL-R10A, HA-NCL-R10F or HA-NCL-R10K as indicated), or not (empty vector). GAPDH was used as a loading control. EBNA1/GAPDH ratios are indicated below the gels. (**C**) The same experiment as in Figure 2, except that RNA pulldowns were performed using extracts from H1299 cells expressing HA-tagged wild-type nucleolin (HA-NCL-wt) or HA-NCL-R10A as indicated. In contrast to HA-NCL-wt which binds to G4 of both EBNA1 and ARPC2 in a G4-dependent manner, only residual binding is observed for HA-NCL-R10A. The blot represents *n* ≥3. (**D**) The same experiment as in (C), except that RNA pulldowns were performed using extracts from H1299 cells expressing HA-tagged wild-type nucleolin (HA-NCL-wt) or HA-NCL-R10F as indicated. Similarly to HA-NCL-wt, HA-NCL-R10F binds to G4 of both EBNA1 and ARPC2 in a G4-dependent manner, albeit less efficiently.

Our results indicate that the binding of nucleolin to G4 of EBNA1 mRNA depends both on its C-terminal RGG motif and on an optimal type I protein arginine methyltransferase (PRMT) activity. The binding of nucleolin to the G4 of EBNA1 mRNA is direct (21). In addition, arginines of RGG motifs are known to be the main substrate of type I PRMT. Therefore, the most likely hypothesis is that type I PRMTs methylate arginines of the C-terminal RGG motif of nucleolin, thereby favouring its interaction with G4 of EBNA1 mRNA. To test this possibility, we again made use of the fact that the yeast nucleolin Nsr1 is not essential and generated a yeast strain that expresses a mutant form of Nsr1 in which all eight arginines of its RGG motif were replaced by alanines (nsr1-R8A) as the sole source of nucleolin. As shown in Figure 6A, this strain (*Y33 nsr1Δ + nsr1-R8A*) behaves like a strain that expresses no nucleolin or only nsr1ΔRGG (*Y33 nsr1Δ + nsr1ΔRGG*) as it forms white colonies, in contrast to the control strain that expresses Nsr1 (*Y33 nsr1Δ + NSR1wt*) which forms pink colonies due to the inhibitory effect of 43GAr on translation of its own mRNA. This is consistent with the possibility that the role of type I PRMT in GAr-based inhibition of translation involves methylation of the arginines of the C-terminal RGG motif of nucleolin, which may in turn regulate the ability of this motif to directly interact with the G4 of EBNA1 mRNA.

We then performed the same type of experiment with the human nucleolin NCL. Using site-directed mutagenesis, we replaced the 10 arginines of the C-terminal RGG motif of HA-tagged NCL (HA-NCL) by either alanine (HA-NCL-R10A), lysine (HA-NCL-R10K) to prevent methylation while maintaining the positive charge, or phenylalanine (HA-NCL-R10F) as a mimic of methylated arginine. Indeed, it is known that methylation of arginine alters charge distribution and increases the volume and π-surface area of the guanidinium moiety, making this residue more hydrophobic and more prone to π-stacking (54,55). Hence it has been shown that phenylalanine may mimic methylated arginine as phenylalanine carries a bulky hydrophobic aromatic moiety (56,57). We overexpressed these various forms of NCL in Raji cells, which are type III latency EBV-infected Burkitt's lymphoma cells, to assess their effect on the endogenous EBNA1 level. Indeed, overexpression of HA-NCL-wt has been shown to lead to a significant decrease in the EBNA1 endogenous level in this cell line, consistent with its role in GAr-based inhibition of translation (21). As shown in Figure 6B, whereas overexpression of wild-type NCL (HA-NCL-wt, second lane) led to a significant decrease in EBNA1 level as compared with GAPDH and with the control with empty plasmid (first lane) as previously observed (21), both HA-NCL-R10A (third lane) and HA-NCL-R10K (fifth lane) had no effect despite being expressed at a similar level as NCL-wt. In contrast, HA-NCL-R10F (fourth lane) led to a decrease in EBNA1 level which is similar to that induced by overexpression of wild-type NCL, suggesting that it is also able to decrease EBNA1 expression. Next we tested the ability of HA-NCL-R10F and HA-NCL-R10A to interact with G4 of EBNA1 mRNA, as compared with HA-NCL-wt. For this, we performed RNA pulldown experiments similar to those described in Figure 2C. In line with the effect of the mutations on EBNA1 expression, we observed that HA-NCL-R10A does not bind to G4 of EBNA1 mRNA (Figure 6C), whereas HA-NCL-R10F does, albeit less efficiently than HA-NCL-wt (Figure 6D). We have also tested the functionality of all the mutants of NCL and Nsr1 in *Y33 nsr1Δ* and found that NCL-R10F and, to a lesser extent, *nsr1-R8F* are able to partially complement the GAr-based inhibition of protein expression in yeast (Supplementary Figure S3). In addition, we determined the cellular localization of HA-NCL-wt, HA-NCL-R10A, HA-NCL-R10F and, as a control, HA-NCLΔRGG, and found that all these forms of NCL are mainly localized in the nucleus (Supplementary Figure S4). All these results are consistent with the possibility that the role of type I PRMT in GAr-based inhibition of translation involves methylation of the arginines of the C-terminal RGG motif of nucleolin.

### Inhibition of human type I protein arginine methyltransferases impacts GAr-based inhibition of antigen presentation

**Figure 7.**
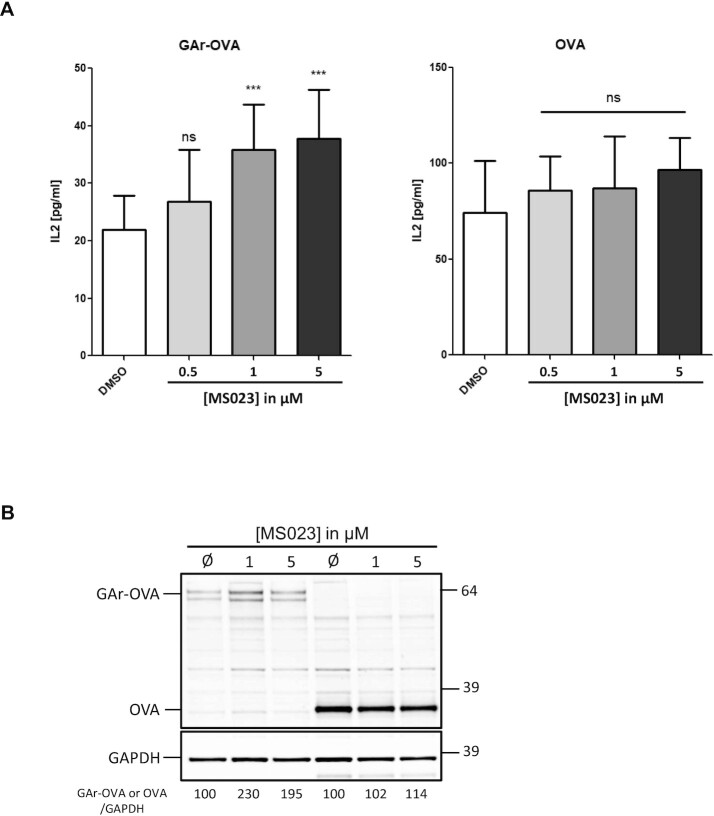
Inhibition of human type I arginine methyltransferases impacts GAr-restricted antigen presentation. (**A**) IL2 concentration (pg/mL) in the supernatant of H1299 cells expressing GAr-OVA (left) or OVA (right) and co-cultured with naive OVA_257–264_ (SIINFEKL peptide)-specific CD8^+^ cells, were determined following their treatment with DMSO (control), or MS023 at various concentrations as indicated. Data are from three biological replicates (***P <0.0001; ns, not significant). (**B**) SDS–PAGE and western blot analysis of cells used in (A).

Our results indicate that type I PRMTs are critically involved in the interaction between NCL and G4 of the GAr-encoding sequence of EBNA1 mRNA. As the GAr-based limitation of both translation and antigen presentation critically depends on this interaction (21), this suggests that human type I PRMTs are therapeutic targets to unveil the oncogenic EBV to the immune system. To test this hypothesis, we determined if inhibition of type I PRMTs by MS023 has an effect on GAr-restricted major histocompatibility complex (MHC) class I antigen presentation. Indeed, as MS023 led to a GAr-dependent increase in protein expression, it was also expected to stimulate GAr-restricted antigen presentation. For this purpose, we determined the effect of MS023 treatment on the GAr-restricted presentation of the ovalbumin-derived antigenic peptide SIINFEKL (OVA_257–264_) complexed to the murine Kb MHC class I receptor using a previously described T-cell assay (21,58). Once complexed to the murine Kb class I receptor, SIINFEKL is specifically recognized by T cells from the B3Z hybridoma, leading to their activation and therefore to production of IL2. Hence, we measured, by ELISA, IL2 produced by B3Z T-hybridoma cells cultured with H1299 cells co-transfected with both Kb expression vector and 235GAr-OVA or, as a control, OVA-expressing plasmids. As previously reported (22), when mixed with B3Z T cells, 235GAr-OVA-expressing H1299 cells led to a much weaker IL2 production (∼22 pg/ml), as compared with OVA-expressing H1299 cells (∼75 pg/ml) (Figure 7A). This is due to the GAr-based limitation of translation and antigen presentation. Strikingly, treatment of cells transiently transfected with 235GAr-OVA with increasing concentrations of MS023 significantly increased the production of IL2 in a dose-dependent manner, whereas no significant effect was observed for OVA-expressing control cells (Figure 7A). These observations are in line with MS023’s effect on GAr-restricted protein expression as MS023 led to a significant increase of GAr-OVA expression, whereas it had no significant effect on OVA expression (Figure 7B). This shows that inhibition of type I PRMTs increases GAr-restricted antigen presentation by MHC-I, suggesting that human type I PRMTs are potential therapeutic targets to unveil the oncogenic EBV to the immune system.

## DISCUSSION

In this paper we first show that the C-terminal RGG motif of NCL is crucial for its interaction with G4 of the GAr-encoding sequence of EBNA1 mRNA. This is fully consistent with recent biophysical studies that assess a role for the RGG motif of NCL in the binding to DNA G4 (32,59). This is also in line with the recently described dependence on the C-terminal RGG of Nsr1 for its role in G4-associated genome instability as well as its ability to bind DNA-G4 (45). In line with this, the N-terminal RGG motif of AVEN has been shown to bind RNA-G4 within the coding region of *MLL1* and *MLL4* mRNA, thereby increasing their polysomal association and translation (60). Interestingly, the role of AVEN was also described to be dependent on arginine methylation (60). Of note, this does not mean that one and/or the other of the various RRM domains of NCL are not important for (or do not participate in) the binding of NCL on RNA-G4.

Next, given that RGG motifs are among the main substrates of type I PRMTs, particularly in yeast, this prompted us to determine if these enzymes may modulate the ability of NCL to interact with G4 of EBNA1 mRNA. Hence we observed that the inhibition of type I PRMTs (by specific inhibitors or by siRNA) in human cells does affect: (i) the ability of NCL to interact with G4 of EBNA mRNA and (ii) the GAr-based inhibition of protein expression. This is also true in yeast, as we observed that the deletion of the *HMT1* gene, which encodes the main yeast type I PRMT1 that accounts for the vast majority of arginine methylation (41) and whose main substrates are RGG motifs (40), interferes with the ability of the yeast nucleolin Nsr1 to interact with G4 of EBNA1 mRNA and also with the GAr-based inhibition of protein expression. This led us to examine if inhibition of type I PRMT1 could interfere with GAr-based limitation of antigen presentation, a mechanism at the root of immune evasion of EBNA1, and thus of EBV. Importantly, we found that inhibition of type I PRMTs by MS023, which was recently described as a specific inhibitor of this family of PRMTs (52), suppresses the GAr-based limitation of antigen presentation in a dose-dependent manner. Hence we have identified type I PRMTs as new druggable therapeutic targets to unveil EBNA1 to the immune system. Of note, type I PRMTs may appear as relatively non-specific therapeutic targets as their inhibition could in principle disturb methylation of many proteins, as has been initially considered for drugs inhibiting protein kinases or phosphatases. However, since RGG motifs are the main substrates of these enzymes, and as, in our various assays, type I PRMT inhibitors did not exhibit significant toxicity at concentration ranges in which they significantly affect both GAr-based translation inhibition and the NCL/G4 of EBNA1 mRNA interaction (Supplementary Figure S5), the lack of specificity may not be an issue.

**Figure 8.**
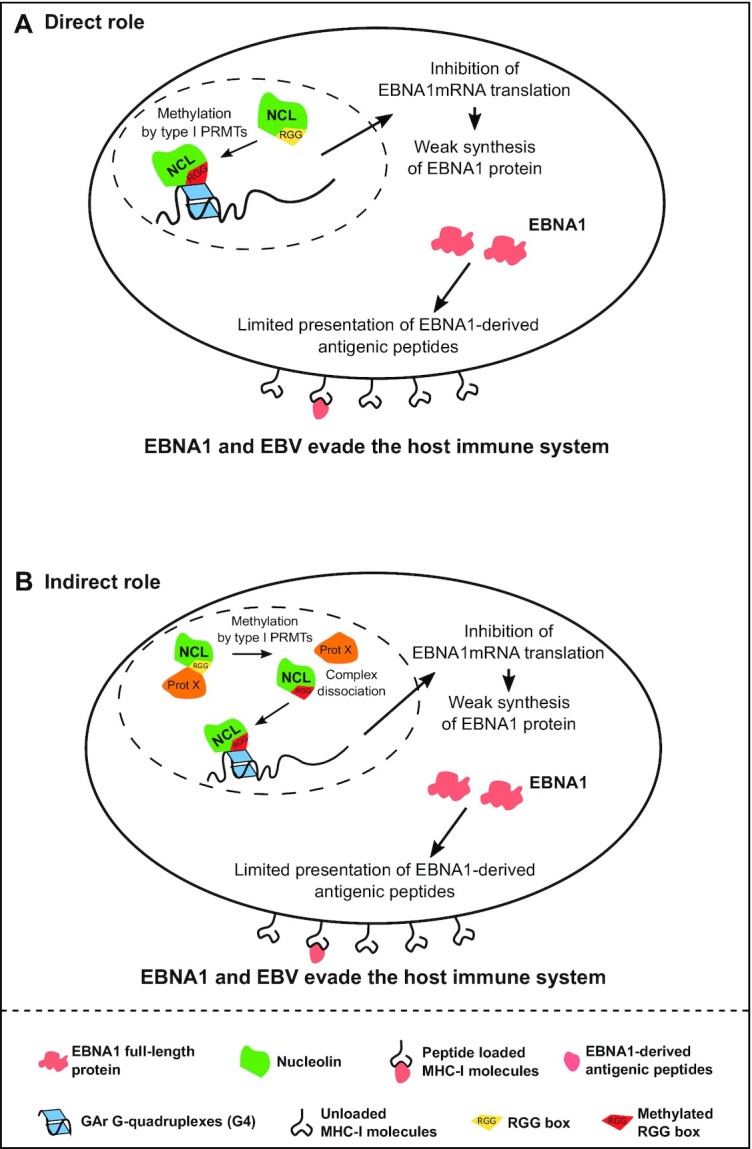
Model. The interaction between NCL and G4 of EBNA1 mRNA is direct and the C-terminal RGG motif of NCL is required for both the ability of NCL to interact with G4 that form in the GAr-encoding sequence of EBNA1 mRNA and for NCL’s crucial role in GAr-based inhibition of translation, the molecular mechanism at the root of EBNA1 immune evasion. RGG motifs are the main substrates of type I PRMTs. The interaction between NCL and G4 of EBNA1 mRNA, as well as the GAr-based inhibition of translation and EBNA1 immune evasion, depend on type I PRMTs, in particular PRMT1. Results of the site-directed mutagenesis of the arginines of the RGG motif of NCL experiments suggest that the role of type I PRMTs in EBNA1 immune evasion involves methylation of the arginines of the RGG motif of NCL. Hence, two possible models for the role RGG methylation by type I PRMTs in EBNA1 evasion can be envisaged: direct (**A**) or indirect (**B**). In the direct model (A), the methylation of arginines of the RGG motif of NCL would directly favour the interaction of the latter with G4 of EBNA1 mRNA and therefore the inhibition of its translation, which in turn allows EBNA1 and EBV to evade the immune system. In the indirect model (B), the non-methylated RGG motif of NCL would be sequestered by a protein partner [protein X, which may be nucleolin itself or another protein such as the various ribosomal proteins which have been shown to interact with the RGG motif of NCL (62)], thereby preventing its interaction with G4 of EBNA1 mRNA. In this model, methylation of the arginines of the RGG motif would interfere with its interaction with protein X, thus releasing NCL and allowing its RGG motif to interact with G4 of EBNA1 mRNA. Our *in vitro* assays did not show any significant difference between methylated and unmethylated RGG for the binding of EBNA1 mRNA G4, suggesting the indirect role is the most likely.

The precise mechanism by which type I PRMTs control EBNA1 immune evasion remains unclear. However, given that RGG motifs represent a major substrate for type I PRMTs, and since we show here that the RGG motif of NCL is crucial for its interaction with G4 of the GAr-encoding sequence of the EBNA1 mRNA [an interaction which is direct (21)], it is tempting to speculate that methylation of arginines in the C-terminal RGG motif of NCL by type I PRMTs favours this interaction. Fully consistent with this possibility is our observation that replacing the eight arginines of the RGG motif of yeast nucleolin Nsr1 by alanines abolishes its ability to participate in GAr-based inhibition of translation. We obtained the same result when replacing the 10 arginines of the C-terminal RGG motif of NCL by alanines or by lysines. Conversely, the replacement of the 10 arginines of the C-terminal RGG motif of NCL by the bulky hydrophobic constitutive methylated arginine-mimetic phenylalanine restored GAr-based inhibition of protein expression, suggesting that π-stacking interactions of the arginines of the RGG motif are likely to be involved in the ability of NCL to interact with G4 of EBNA1 mRNA and that these interactions might be reinforced by arginine methylation. Indeed, arginines are known to establish preferential interactions with guanine rings through complex interactions involving π-cation, π-π stacking and H-bonding (61). Hence it makes sense that arginine–guanine pairs are determinant for binding to the external guanine quartets of G4 structures which are recognition elements for G4-binding proteins (32,59). In line with this, we observed that inhibition of type I PRMTs (by small molecular weight inhibitors or by siRNA) interferes with the ability of both yeast and human nucleolin to bind to G4 of EBNA1 mRNA, as shown by RNA pulldown experiments and PLAs. Postulating that methylation of the arginines of the RGG motif of nucleolin is crucial for the role of nucleolin in GAr-based inhibition of translation, the question is now to determine if the role of RGG methylation is direct or indirect. In other words and as shown in Figure 8, is the methylation of various arginines directly involved by physically promoting the interaction between the C-terminal RGG motif of NCL and G4 of EBNA1 mRNA, or does it plays its role by preventing the interaction of NCL with other partner(s), thereby releasing NCL which is then free to interact with G4 of EBNA1 mRNA? In favour of the first possibility (‘direct’ role of RGG methylation) data are available that show that RGG methylation may interfere with its ability to interact with some of its partners (that can be proteins, DNA or RNA) (34). In favour of the second possibility (‘indirect’ role of RGG methylation) are the recent observations that Scd6, a yeast protein involved in translation inhibition, is able to self-associate via its RGG motif and that this self-association prevents its translation repression activity and is negatively regulated by Hmt1-dependent methylation (33). To test these two possibilities (‘direct’ or ‘indirect’ role of RGG methylation), we have performed two types of *in vitro* experiments. We first performed *in vitro* arginine methylation of bacterially produced recombinant NCL (hence being initially non-methylated) and then assessed its ability to bind G4 of EBNA1 mRNA, as compared with non-methylated recombinant NCL. The result of this new experiment is shown in Supplementary Figure S6. We found no significant difference between methylated and non-methylated NCL for binding to the EBNA1 mRNA sequence forming one G4 (18-mer) and a slightly stronger binding of methylated NCL when a two-repeat EBNA1 mRNA sequence forming two G4 (36-mer) was used as a binding partner. This suggests that the effect of arginine methylation on the ability of the C-terminal RGG motif of NCL to bind G4 of EBNA1 mRNA could be indirect, by interfering with the interaction of the RGG motif with unknown partner(s) whose binding may prevent the RGG motif from interacting with G4 of EBNA1 mRNA. The factor interacting with the RGG motif of NCL could be a ribosomal protein as NCL has been reported to interact with several ribosomal proteins through its RGG motif (62). Alternatively, the binding partner could be NCL itself as it has been reported to self-associate (63). However, in this case, this self-interaction rather involves NCL’s central RRM domains, and is therefore less likely to prevent its C-terminal RGG motif from interacting with G4 of EBNA1 mRNA. In line with this, using two-hybrid, we were not able to observe self-interaction for either yeast (Nsr1) or human (NCL) nucleolin, even in a *hmt1Δ* strain (G.A. and M.B., unpublished observations).

Of note, one caveat with *in vitro* arginine methylation of NCL is that the extent of methylation is difficult to control. Hence, we obtained a heterogeneous mixture of NCLs harbouring various degrees of methylation (on average eight arginines per protein featuring mono-methylation, or symmetrical or asymmetrical di-methylations, Supplementary Figure S7) which may not reflect the biological situation. To overcome this limitation, we have also performed another *in vitro* experiment based on two NCL RGG peptides—one being asymmetrically di-methylated on the 10 arginines, while the other was non-methylated. We used these two peptides to determine if arginine methylation may have a direct effect on G4 binding. We found that both peptides display a similarly high capacity to bind G4 of EBNA1 mRNA (*k*_D_ in the nM range with a slight difference in favour of the methylated peptide, Supplementary Figure S8), which is fully in line with the results of the experiment using the recombinant protein. Taken together, these *in vitro* data suggest that the effect of arginine methylation on G4 binding is most probably indirect as methylation only displays a marginal effect on the *in vitro* direct interaction between G4 and the RGG motif of NCL. Importantly, the identification of the putative binding partner of the NCL RGG motif that sequesters it, thereby preventing its interaction with G4 of EBNA1 mRNA, is an important aspect that has not been solved in the present study. Given the central role of NCL in RNA biology, the identification of this factor should be the subject of future studies. This will be an important step as, beyond its involvement in EBNA1 immune evasion, this mechanism may represent an original and general way to regulate the ability of NCL to interact with RNA.

Apart from NCL, which is the first host factor crucially involved in GAr-based inhibition of translation and of antigenic peptide production, an important question is to determine if other proteins are involved in this process, through binding to G4 of EBNA1 mRNA like NCL. Interestingly, EBNA1 itself contains two RGG motifs: one upstream of GAr and one directly downstream, and these two RGG motifs have been shown to bind RNA-G4 (64), leaving the possibility that EBNA1 may contribute to the limitation of its own expression by binding to G4 of its own mRNA. Importantly, this putative role of EBNA1 could also be controlled by type I PRMTs.

Finally, the deimination of arginine into citrulline, a process called citrullination and which is catalysed by peptidylarginine deiminases, prevents their methylation. Interestingly, it has been reported that the infection by EBV activates several PRMTs, including PRMT1, and inactivates PADI4, the main peptidylarginine deiminase (65). This is fully in line with the results reported here and it is tempting to speculate that the infection by EBV, by activating PRMT1 and inactivating PADI4, favours the methylation of the RGG motif of NCL (and possibly of other RGG motif-containing proteins such as EBNA1), thereby increasing their ability to bind to G4 of EBNA1 mRNA, which in turn leads to inhibition of EBNA1 mRNA translation, thus limiting the production of EBNA1-derived antigenic peptides, ultimately leading to immune evasion of EBV.

The precise and comprehensive role of type I PRMTs in GAr-based inhibition of translation, a molecular mechanism at the root of EBNA1 immune evasion and potentially another role for NCL in RNA biology, clearly deserves deeper investigations and will be the subject of future studies. Importantly, whatever the precise mechanism (see Figure 8), our present work unambiguously defines type I PRMTs as therapeutic targets to unveil EBV-infected cells to the immune system, thereby providing new potential therapeutic avenues to unveil EBV-related cancers to the immune system.

## Supplementary Material

gkac915_Supplemental_Files
