# Supplementary material for: Type I arginine methyltransferases are intervention points to unveil the oncogenic Epstein-Barr virus to the immune system
Source: Nucleic Acids Res. 2022 Nov 9;50(20):11799–819. doi: 10.1093/nar/gkac915 (PMC9723642; doi:10.1093/nar/gkac915)
Supplement: gkac915_Supplemental_Files [file gkac915_supplemental_files.zip › Supplementary_Figure_1_Angrand_et_al_revised.pdf]

Supplementary Figure 1

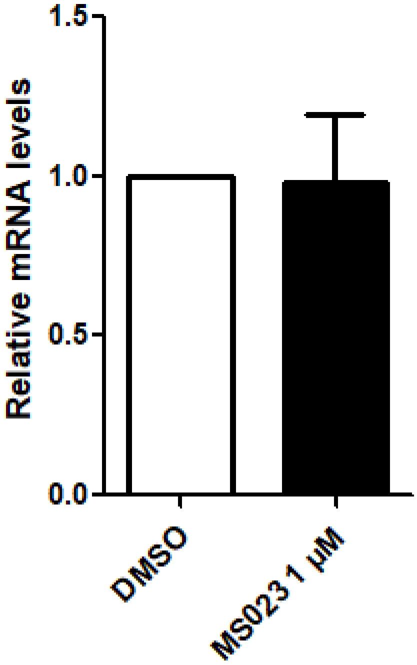

Treatment with MS023 has no effect on endogenous EBNA1 mRNA levels in Mutu-1 cells. The relative expression level of EBNA1 mRNA was determined by quantitative RT-PCR and calculated using the ΔΔCt method with control GAPDH. Three biological replicates were performed.
