# Supplementary material for: Type I arginine methyltransferases are intervention points to unveil the oncogenic Epstein-Barr virus to the immune system
Source: Nucleic Acids Res. 2022 Nov 9;50(20):11799–819. doi: 10.1093/nar/gkac915 (PMC9723642; doi:10.1093/nar/gkac915)
Supplement: gkac915_Supplemental_Files [file gkac915_supplemental_files.zip › Supplementary_Figure_2_Angrand_et_al_revised.pdf]

Supplementary Figure 2

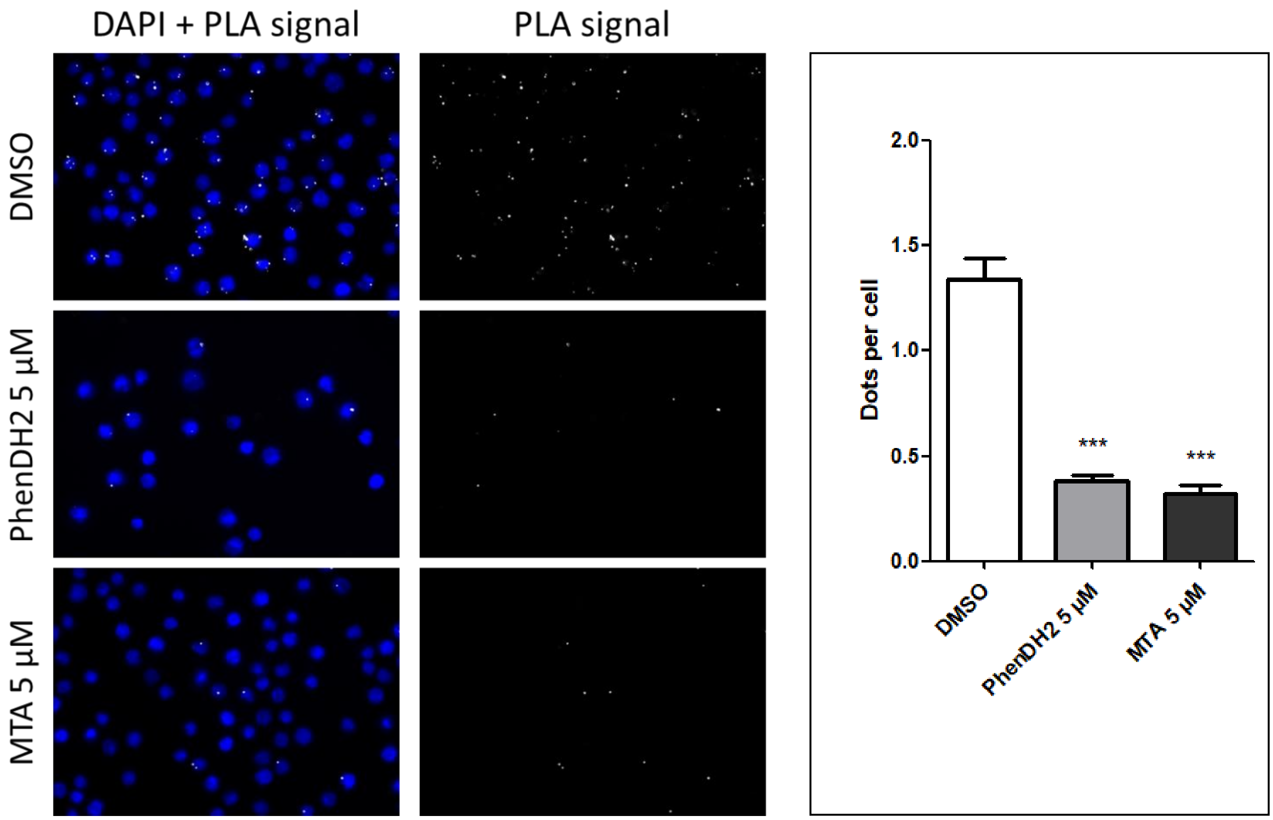

As MS023, MTA, another PRMT inhibitor, interferes with the interaction between NCL and G4 of EBNA1 mRNA. Adaptation of the proximity ligation assay (PLA) to monitor protein/RNA interaction performed in Mutu-1 cells natively expressing EBNA1. Left and middle panels: microscopy images of cells treated with DMSO (compound vehicle, control), PhenDH2 (5 µM, a G4 ligand which has been shown to interfere with the interaction between NCL and G4 of EBNA1 mRNA (Reznichenko O et al *Eur J Med Chem* 2019) used as a control) or MS023 (5 µM) as indicated. Nuclei were revealed by DAPI staining and appear in blue; white dots (PLA signals) indicate interaction between NCL and G4 of EBNA1 mRNA. Right panel: number of nuclear PLA signals (dots) per cell in Mutu-1 cells treated with DMSO (control) or with PhenDH2 (5 µM) or MTA (5 µM). Data from two biological replicates, 200 cells per sample were compared using ANOVA in conjunction with Tukey's test using GraphPad Prism 5 for Windows (GraphPad Software) (***, $p < 0.0001$).
