# Supplementary material for: Type I arginine methyltransferases are intervention points to unveil the oncogenic Epstein-Barr virus to the immune system
Source: Nucleic Acids Res. 2022 Nov 9;50(20):11799–819. doi: 10.1093/nar/gkac915 (PMC9723642; doi:10.1093/nar/gkac915)
Supplement: gkac915_Supplemental_Files [file gkac915_supplemental_files.zip › Supplementary_Figure_3_Angrand_et_al_revised.pdf]

Supplementary Figure 3

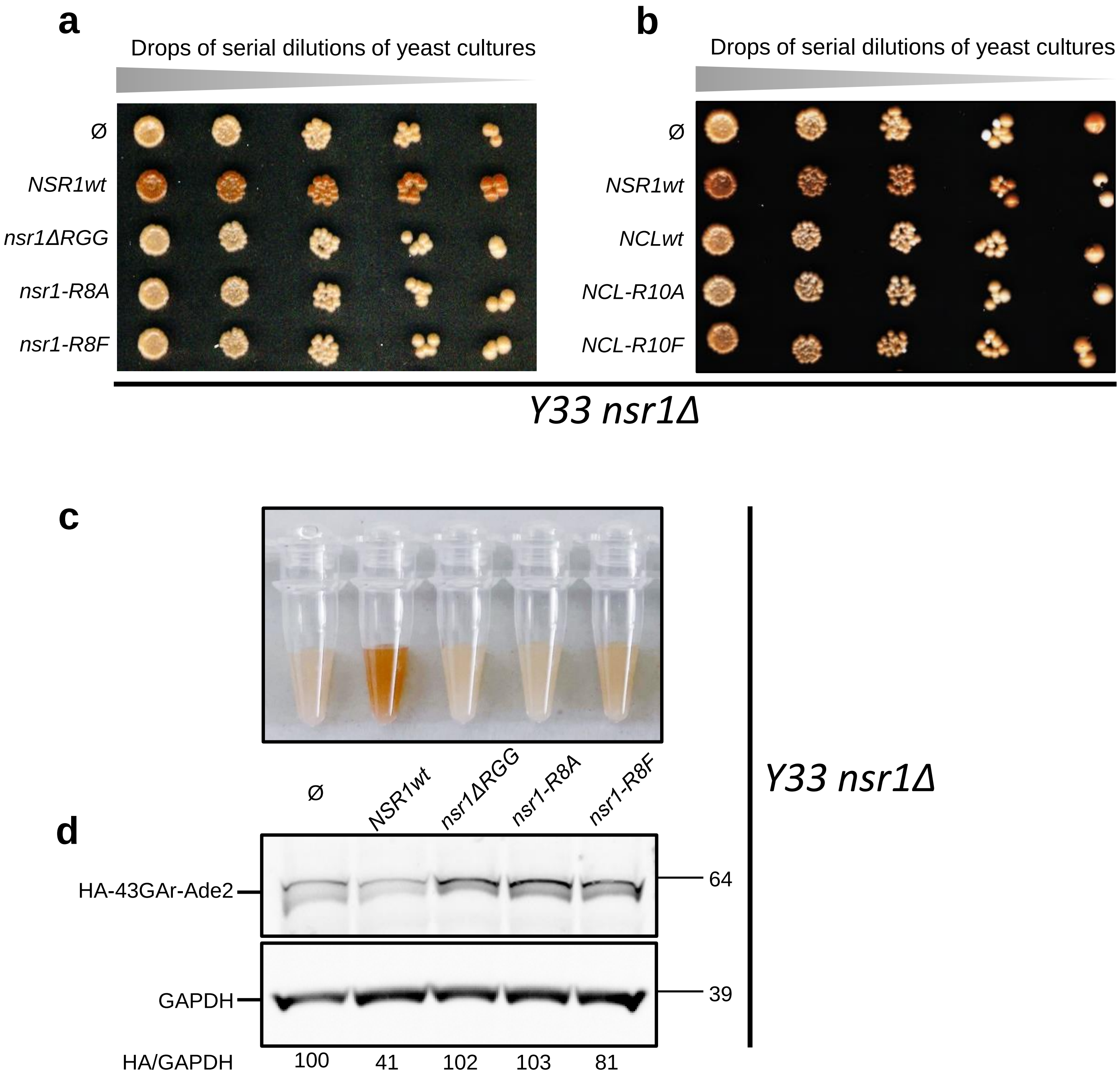

Analysis of the ability various forms of Nsr1 (**a**) or NCL (**b**) to complement the role of Nsr1 in GAr-based inhibition of translation in an *Y33 nsr1Δ* strain, as compared to the wild type Nsr1 (*NSR1wt*, second lines). Serial dilutions of the various transformed strains were spotted onto agar-based solid medium. The colour of yeast colonies is used as the readout. (**c**) Same strains as in (**a**) grown in liquid minimal medium to better visualize the pink shades. (**d**) Western blot analysis of the level of HA-43GAr-Ade2 in the same strains as in (**a**). HA-43-GAr-Ade2/GAPDH ratios are indicated below the gels.
