# Supplementary material for: Type I arginine methyltransferases are intervention points to unveil the oncogenic Epstein-Barr virus to the immune system
Source: Nucleic Acids Res. 2022 Nov 9;50(20):11799–819. doi: 10.1093/nar/gkac915 (PMC9723642; doi:10.1093/nar/gkac915)
Supplement: gkac915_Supplemental_Files [file gkac915_supplemental_files.zip › Supplementary_Figure_4_Angrand_et_al_revised.pdf]

Supplementary Figure 4

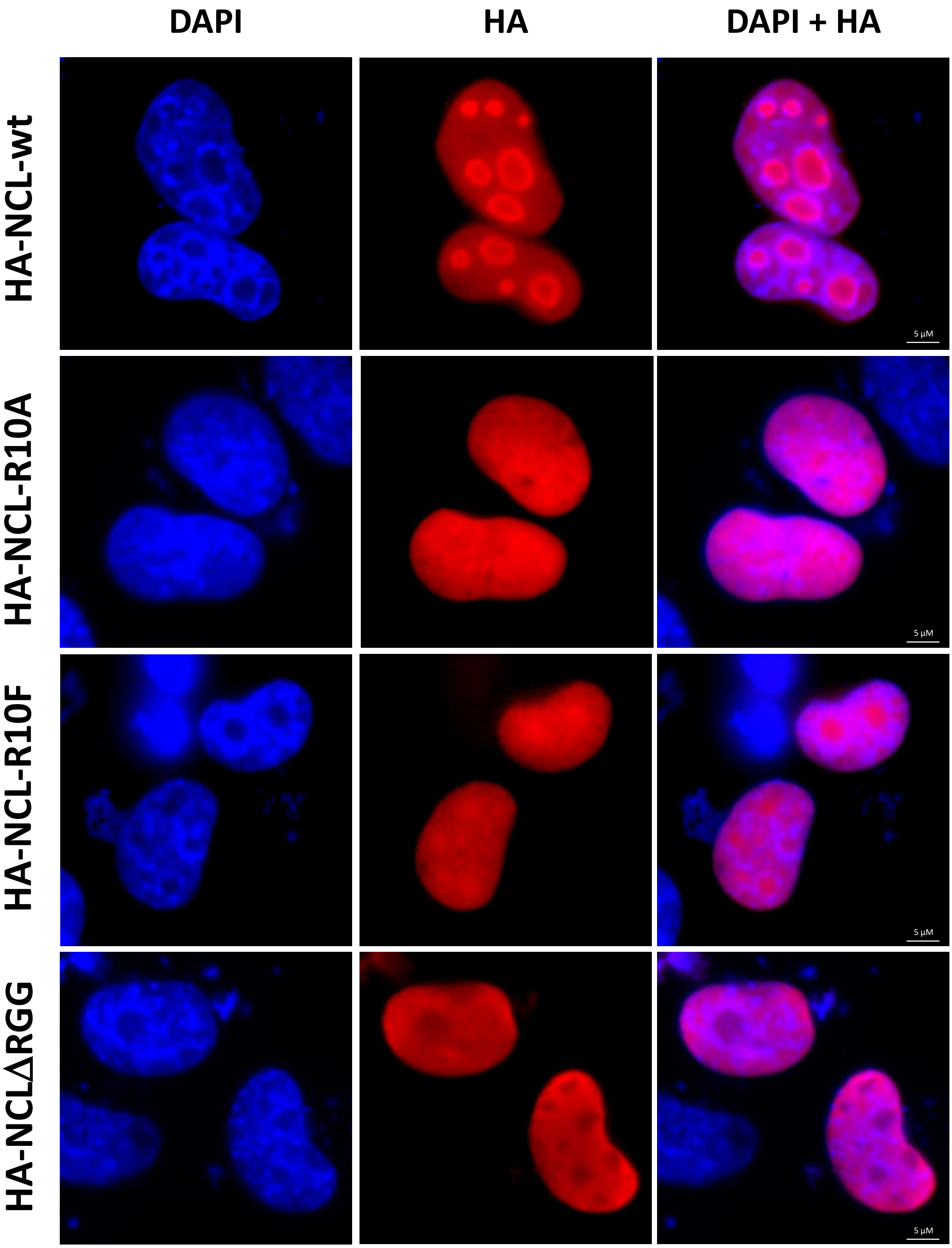

Cellular localization of the various NCL mutants used. Immunofluorescence (IF) was performed in H1299 cells transfected with HA-NCL-wt, HA-NCL-R10A, HA-NCL-R10F or HA-NCLΔRGG using a mouse anti-HA antibody. Note that the three mutants of NCL remain localized in the nucleus but are less concentrated in the nucleolus than NCLwt.
