# Supplementary material for: Type I arginine methyltransferases are intervention points to unveil the oncogenic Epstein-Barr virus to the immune system
Source: Nucleic Acids Res. 2022 Nov 9;50(20):11799–819. doi: 10.1093/nar/gkac915 (PMC9723642; doi:10.1093/nar/gkac915)
Supplement: gkac915_Supplemental_Files [file gkac915_supplemental_files.zip › Supplementary_Figure_5_Angrand_et_al_revised.pptx]

## Slide 1
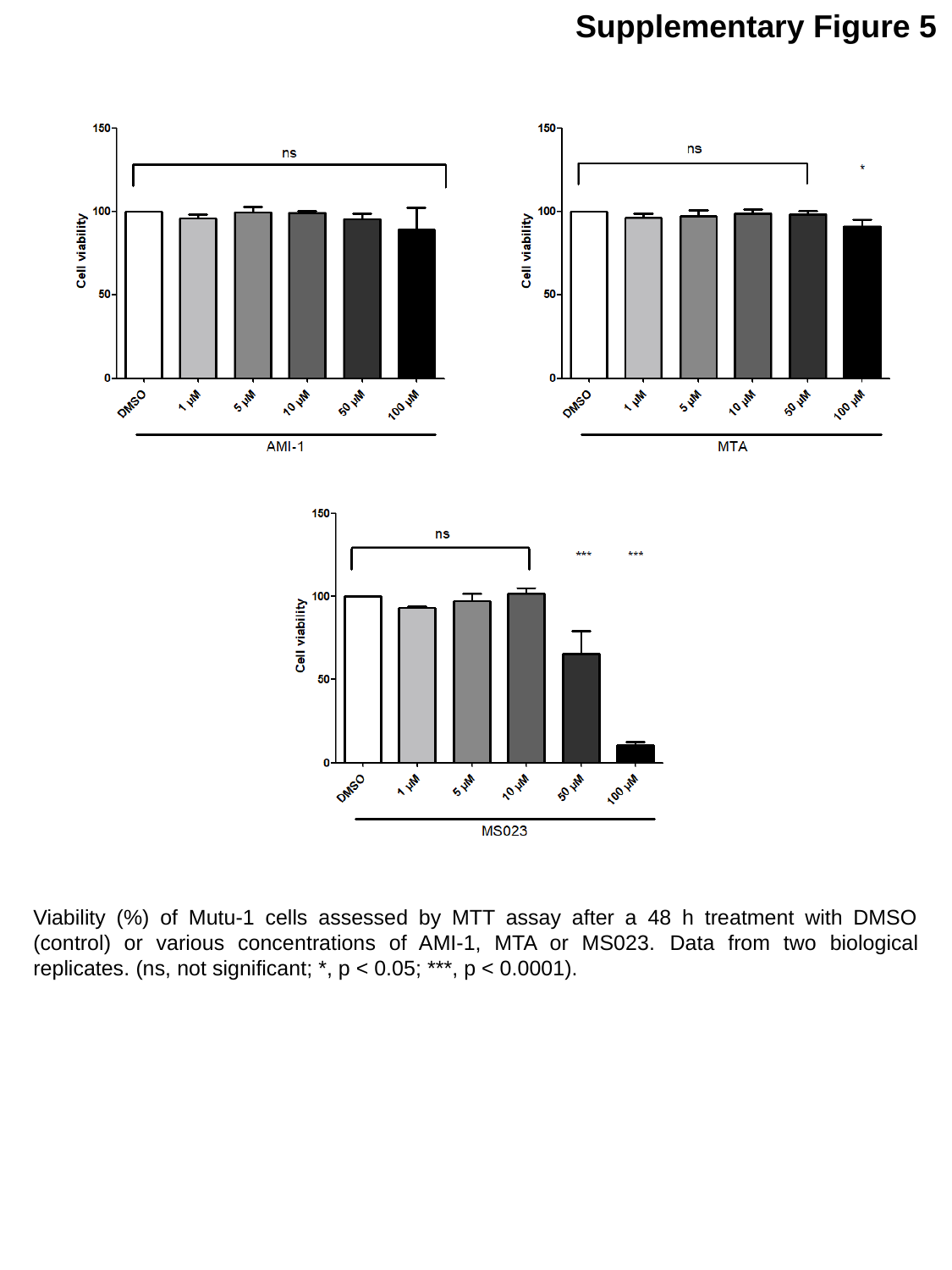

Supplementary Figure 5
Viability (%) of Mutu-1 cells assessed by MTT assay after a 48 h treatment with DMSO (control) or various concentrations of AMI-1, MTA or MS023. Data from two biological replicates. (ns, not significant; *, p < 0.05; ***, p < 0.0001).
