# Supplementary material for: Type I arginine methyltransferases are intervention points to unveil the oncogenic Epstein-Barr virus to the immune system
Source: Nucleic Acids Res. 2022 Nov 9;50(20):11799–819. doi: 10.1093/nar/gkac915 (PMC9723642; doi:10.1093/nar/gkac915)
Supplement: gkac915_Supplemental_Files [file gkac915_supplemental_files.zip › Supplementary_Figure_6_Angrand_et_al_revised.pdf]

Supplementary Figure 6

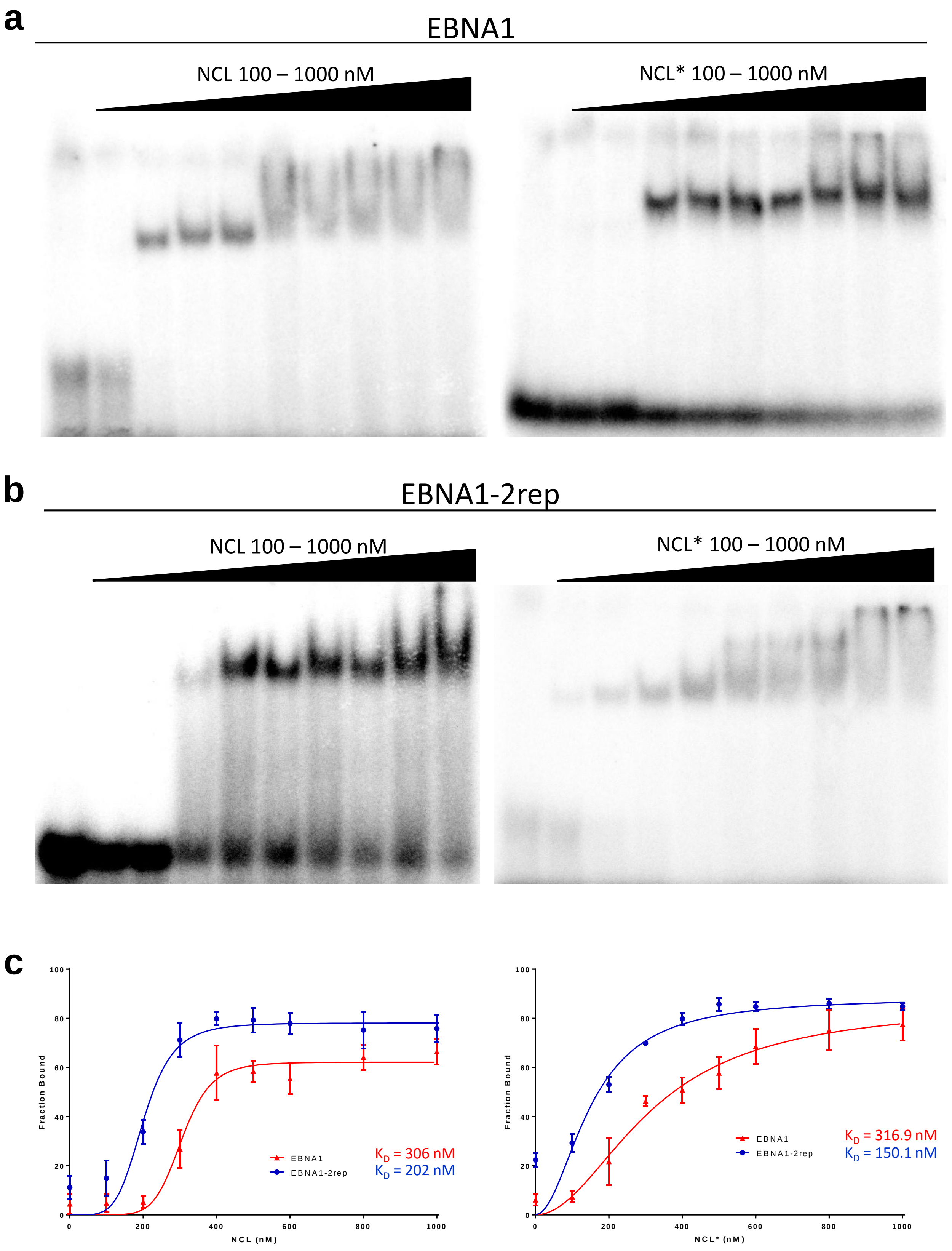

Electrophoretic mobility shift assay (EMSA) of the binding of NCL and methylated NCL (NCL*) to EBNA1 (**a**) and EBNA1-2 repeat (**b**). Lower band correspond to free RNA, and higher band indicate RNA-NCL or RNA-NCL* complex. (**c**) Binding curves obtained from quantification of bound NCL and methylated NCL (NCL*) as a function of concentration (average of triplicate experiments).
