# Supplementary material for: Type I arginine methyltransferases are intervention points to unveil the oncogenic Epstein-Barr virus to the immune system
Source: Nucleic Acids Res. 2022 Nov 9;50(20):11799–819. doi: 10.1093/nar/gkac915 (PMC9723642; doi:10.1093/nar/gkac915)
Supplement: gkac915_Supplemental_Files [file gkac915_supplemental_files.zip › Supplementary_Figure_7_Angrand_et_al_revised.pdf]

Supplementary Figure 7

a

b

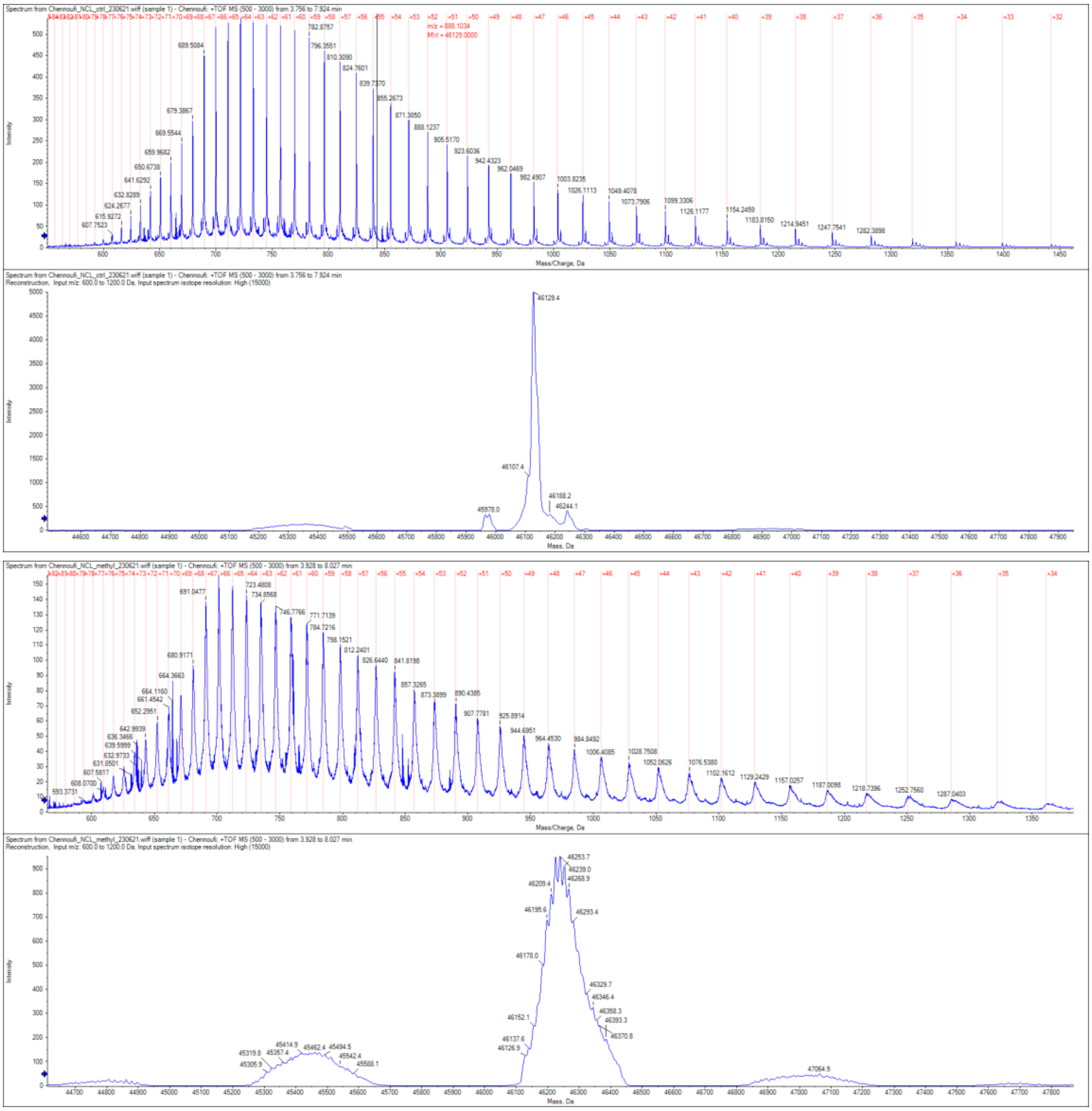

c

284

AKQKAAPEAKKQKVEGTEPTTAFNLFVGNLNFNKSAPELKTGISDVFAKNDLAVVDVRIGMTRKFGYVDFE

SAEDLEKALELTGLKVFGNEIKLEKPKGKDSKKERDARTLLAKNLPYKVTQDELKEVFEDAAEIRLVSKDGKSK

GIAYIEFKTEADAEKTFEEKQGTEIDGRSISLYYTGEKGQNQDYRGGKNSTWSGESKTLVLSNLSYSATEETL

QEVFEKATFIKVPQNQNGKSKGYAFIEFASFEDAKEALNSCNKREIEGRAIRLELQGPRGSPNARSQPSKTLF

VKGLSEDTTEETLKESFDGSVRARIVTDRETGSSKGFGFVDFNSEEDAKAAKEAMEDGEIDGNKVTLDWAK

PKGEGGFGGRGGGRGGFGGRGGGRGGRGGFGGRGRGGFGGRGGFRGGRGGGGDHKPQGKKTKFE

RGG domain

709

R341, R346, R389, R392, R419, R456, R473, R545, R550, R553, R560, R566, R597, R599, R604,

R655, R659, R665, R669, R672, R678, R680, R686, R690, R693

Mass spectrometry analysis of in vitro NCL methylation. ESI-MS analysis of recombinant NCL before (**a**) and after methylation by PRMT1 (**b**). The broad profile of methylated NCL indicates high heterogenity of the Arg methylation (average of 8 methylated residues). (**c**) Identification of methylated Arg after digestion and nanoLC-MS/MS analysis of resulting peptides. Monomethylated Arg (yellow), dimethylated Arg (blue) and mono or dimethylated Arg (green).
