# Supplementary material for: Type I arginine methyltransferases are intervention points to unveil the oncogenic Epstein-Barr virus to the immune system
Source: Nucleic Acids Res. 2022 Nov 9;50(20):11799–819. doi: 10.1093/nar/gkac915 (PMC9723642; doi:10.1093/nar/gkac915)
Supplement: gkac915_Supplemental_Files [file gkac915_supplemental_files.zip › Supplementary_Figure_8_Angrand_et_al_revised.pdf]

Supplementary Figure 8

a

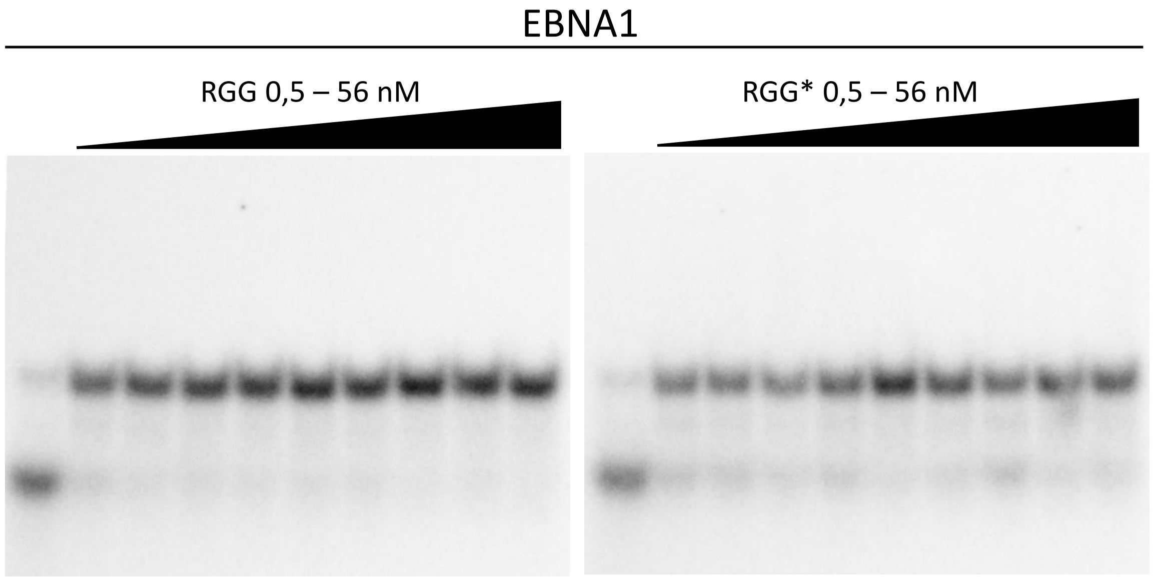

b

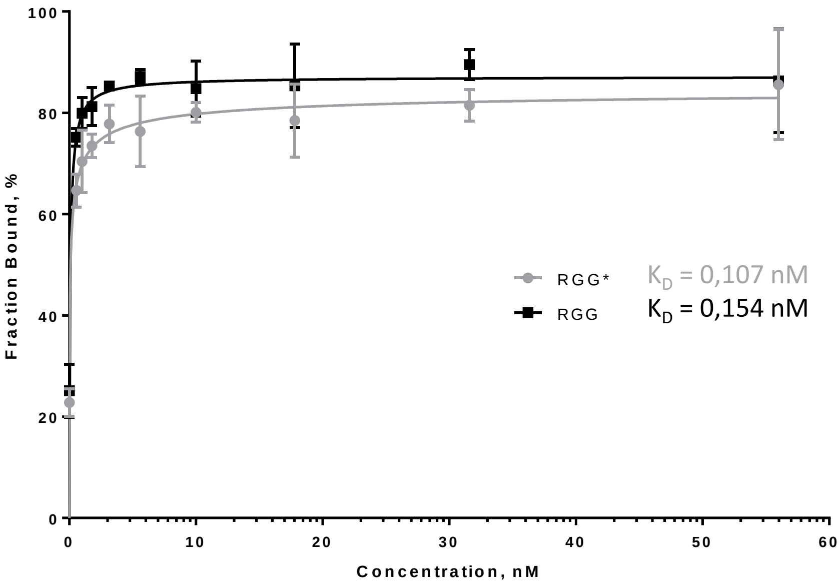

Electrophoretic mobility shift assay (EMSA) of the binding of RGG peptide and methylated RGG peptide (RGG*) to EBNA1 (**a**). Lower band correspond to free RNA, and higher band indicate RNA-RGG or RNA-RGG* complex. (**b**) Binding curves obtained from quantification of bound RGG peptide and methylated RGG peptide (RGG*) as a function of concentration (average of triplicate experiments).
